# Conducting Evaluations in the Context of Tertiary Prevention of Youth Crime: Reflections from the Youth Endowment Fund

**DOI:** 10.3390/bs16050626

**Published:** 2026-04-22

**Authors:** Daniel K. Acquah, Claryn S. J. Kung, Rain M. Sherlock

**Affiliations:** Youth Endowment Fund, London EC2A 3QR, UK; daniel.acquah@youthendowmentfund.org.uk (D.K.A.); rain.sherlock@youthendowmentfund.org.uk (R.M.S.)

**Keywords:** tertiary prevention, youth crime, impact evaluation, randomised controlled trials, intervention adaptation, multi-site trials, race equity, informed consent, recruitment, retention

## Abstract

Serious youth violence is a public health issue nationally in the UK and internationally. The Youth Endowment Fund (YEF) was established in March 2019, with a £200 million endowment and a ten-year mandate, with a mission to prevent children and young people from becoming involved in violence. This article gives an overview of YEF’s successes and challenges to date, focusing specifically on the experience of evaluating tertiary interventions. After providing an overview of YEF’s approach to funding and evaluation, the article summarises YEF’s work focused on tertiary prevention, including: work to test interventions already being implemented in the UK; adapting and evaluating evidence-based interventions from other jurisdictions in the UK; innovations in a group approach to carrying out evaluations; and embedding a focus on racial equity in tertiary prevention. Next, the article discusses the design issues involved in high-quality evaluation of tertiary prevention, including the scale required and the processes for obtaining consent from young people to participate in evaluations. The article then documents the many challenges and lessons learned from implementing tertiary prevention evaluations, especially focusing on the recruitment and retention of young people. Finally, the article discusses the lessons and places them in a wider context.

## 1. Introduction

Serious youth violence is a public health issue nationally in the UK and internationally (Kane et al., 2025; Krug et al., 2002; Youth Endowment Fund, 2024a). The impetus for this Special Issue is the premise that there is a paucity of evidence-based programmes for those who are engaged in the most serious, violent, and often organised, criminal behaviour (tertiary intervention). This article is based on the experience of the Youth Endowment Fund, which was set up, in part, to address the gap in the evidence base. The rest of the article is structured as follows. Section 2 introduces the Youth Endowment Fund’s approach to intervention funding and evaluation. Section 3 summarises YEF’s work focused on tertiary prevention, including: testing interventions already implemented in the UK; adapting and evaluating evidence-based interventions from other jurisdictions in the UK; innovations in group-based evaluation approaches; and embedding a focus on racial equity in tertiary prevention. Section 4 discusses the design issues involved in high-quality evaluation of tertiary prevention, including the scale required and the processes for obtaining consent from young people to participate in evaluations. Section 5 documents the many challenges and lessons learned from implementing tertiary prevention evaluations, especially regarding the recruitment and retention of young people. Section 6 discusses the lessons and places them in a wider context.

## 2. The UK Youth Endowment Fund’s Approach to Funding and Evaluation

The Youth Endowment Fund (YEF) was established in 2019 with a £200 million endowment from the UK Home Office. It is an independent charity and part of the UK government’s What Works Centres, which aim to improve the way government and other public sector organisations create, share and use high-quality evidence in decision-making (Bristow et al., 2016; Vallance et al., 2025). The mission of YEF is to prevent children and young people from becoming involved in violence by finding out what works and building a movement to put this knowledge into practice. Large-scale experimental evaluation in youth justice remains relatively rare in the UK, with persistent challenges around recruitment, attrition and implementation (Axford & Humayun, 2026; Farrington, 1996). Within this context, the YEF has commissioned almost 100 evaluations over the last seven years, of which 60 are impact evaluations, most of which are randomised controlled trials.

The YEF’s approach to funding and evaluation is inspired by both practice in clinical trials and social policy evaluation, including the work of the ‘sister’ What Works Centre for education, the Education Endowment Foundation (Edovald & Nevill, 2021). YEF’s funding and evaluation work is organised through seven essential sectors: youth justice, education, children’s services, health, policing, youth sector, and neighbourhoods. Typically, projects are funded through thematic, sector-specific funding rounds. Key features of YEF’s approach to evaluation include:

- Evaluator panel: YEF commissions independent organisations to rigorously evaluate the implementation and impact of all the interventions it funds. The evaluator panel comprises UK-based higher education institutions and other research organisations (Youth Endowment Fund, n.d.).
- Focus on impact but scope for earlier stage evaluations: As a What Works Centre committed to robust impact evaluation, most evaluations commissioned are randomised controlled trials that seek to establish the causal impact of interventions. We know from trials conducted in the US and in other social policy sectors that these types of evaluation have a low success rate (Epstein & Klerman, 2012; White, 2019). Given that the UK does not have a strong track record of investment in evaluations of youth crime reduction, we recognised the need for early-stage feasibility studies (testing aspects of the intervention) and pilot studies (testing aspects of the evaluation) to develop the pipeline of interventions for impact evaluation.
- Measurement informed by an Outcomes Framework: The primary outcome for each YEF evaluation is selected based on advances in our understanding of the risk and protective factors of young people’s involvement in violence, whilst also aimed at informing the design of prevention programmes that can help reduce young people’s interactions with the criminal justice system.
  ○ Police-recorded crime: This is used as the primary outcome wherever feasible, especially for tertiary prevention. YEF has also produced guidance for how the data can be accessed and used for evaluation purposes (Ellison & Cook, 2025).
  ○ Intermediate risk and protective factors: A robust understanding of risk and protective factors is foundational to the design, implementation, and evaluation of youth violence prevention programmes (Rooney et al., 2024). An established body of research shows that youth violence arises from the interaction of multiple factors across the socioecological spectrum—individual, family, community, and societal (e.g., Baidawi & Ball, 2023; Farrington et al., 2021, 2025; Kleeven et al., 2025; Kowalski et al., 2023; Liu et al., 2024; Peck et al., 2022; Tura et al., 2025; Walker et al., 2022). YEF commissioned a review of systematic reviews (Ullman et al., 2024) to look at the factors most predictive of later crime and violence, drawing on earlier evidence (Caridade & Braga, 2020; de Ribera et al., 2019; Haylock et al., 2020). Together with the input of an expert reference group, this led to our ‘Outcomes Framework’, which is a list of potential primary outcomes that could be chosen in YEF evaluations (Anna Freud Centre, 2022). This body of research continues to grow (e.g., Lambie et al., 2026; Shamrova et al., 2025; Themelidis & Lorenzo-Dus, 2025), and YEF continues to look for ways to make it accessible to practitioners and evaluators.
  ○ Database of measures: A second project (Anna Freud Centre, 2023) aimed to locate and assess the self-reported measurement tools that exist to measure the outcomes in the Outcomes Framework. Given the volume of potential measures, the project thoroughly evaluated them for accessibility and psychometric quality. This database was intended to support YEF and evaluators in making informed decisions about the most suitable outcome measures for each project.
- A commitment to longitudinal follow-up: Not every evaluation collects data on offending, and those that do are often constrained by the evaluation length, considering the time lag between many interventions aimed at crime reduction and actual rates of offending or reoffending. Consequently, the YEF has created a data archive to support reanalysis of trial data. This involved complex legal and technical work with evaluation teams, the Office of National Statistics, the Department for Education and YEF’s legal team. The technical details of the archive are described elsewhere (Youth Endowment Fund, 2023). Here we summarise the three principal uses of the archive:
  ○ Perform quality assurance checks, methodological exploration and further detailed analysis on the published results from our original evaluations.
  ○ Provide descriptive analysis of the data pooled across our projects to better understand the populations at risk of violence.
  ○ Conduct longitudinal follow-up evaluation on the future outcomes of project participants.
- Commitment to open and transparent prevention science: As a major funder, YEF recognises its potential role in improving the commitment to open and transparent practice in prevention science (Buckley et al., 2022; Grant et al., 2022). All YEF studies must produce a pre-registered protocol. Similarly, a statistical analysis plan (SAP) is required, which pre-specifies the analyses the authors intend to carry out. Any deviations from these plans must be carefully documented.
- Robust peer review systems: Following accepted standards in research, all statistical analysis plans and research reports are peer reviewed. One important component of this review is ensuring that reports follow the methods and analyses outlined in the published protocol and the SAP. YEF is also following developments in artificial intelligence (e.g., Naddaf, 2025) especially as a tool for increasing the rigour of detecting protocol deviations, given the known issues in other fields of investigations (Poorolajal, 2025).
- Obligation to publish regardless of results: Another distinctive feature of YEF’s approach is the commitment to publish everything, irrespective of results. This is different to many other funders or publishers of research. The issues with publication bias have been well documented (e.g., Van Aert et al., 2019; Axford et al., 2022a); therefore, the obligation to publish every evaluation report, regardless of the findings, is part of a wider movement to ensure null results in general are published (e.g., Munafò & Neill, 2016).

## 3. Summary of YEF’s Work Focused on Tertiary Prevention

YEF’s funding and evaluation work on tertiary prevention has involved several strands. One has been to evaluate the impact of existing prevention programmes already being implemented in England and Wales (see Section 3.1). A second strand has been to ‘import’ interventions shown to be effective elsewhere and adapt them to the UK context (see Section 3.2). A third strand has focused on methodological innovation to enable a broader range of projects to be evaluated using high-quality experimental methods (see Section 3.3). A fourth aspect of YEF’s work has been to ensure we take a racial and ethnic equity perspective throughout our evaluation work, which we illustrate with reference to a case study of a boxing trial to reduce youth offending (see Section 3.4).

Informing all of these efforts is a significant body of international literature on what works to prevent youth crime and violence, including recent systematic reviews and meta-analyses (e.g., Farrington et al., 2017, 2022; Farrington & Welsh, 2003; Kovalenko et al., 2022; Matjasko et al., 2012; Piolanti et al., 2022; Sherman et al., 2015; Strang et al., 2013; Welsh & Farrington, 2007; Wilson & Lipsey, 2024; Wong et al., 2024). This body of work situates YEF’s work within a wider ecosystem of intervention development, implementation, and evaluation (e.g., Nutley et al., 2009; Oliver et al., 2014). Similar to other evidence-oriented funders, such as the Education Endowment Foundation (Edovald & Nevill, 2021) and the National Institute for Health and Care Research (Dorling et al., 2015), YEF supports interventions at multiple stages of the evidence pipeline, following established models of early-stage development and feasibility testing through to rigorous impact evaluation (Dorling et al., 2015; Gottfredson et al., 2015; Skivington et al., 2021). YEF is also committed to evaluating interventions already in routine use, including those that have not been rigorously specified or tested. This reflects a commitment to generating evidence about real-world practice, including identifying ineffective or potentially counterproductive approaches (Axford et al., 2022b; Halpern, 2015; White, 2019). This approach necessarily involves testing interventions with varying levels of prior specification and evidential support, including those that might otherwise persist in practice without scrutiny. Evaluating such interventions, where uncertainty about effectiveness is a reason for rigorous evaluation is critical to avoid the continued use of untested or ineffective provision within the system (Macintyre, 2011).

### 3.1. Testing Interventions Already Available in the UK

A key pillar of YEF’s work in tertiary prevention was the ‘Another Chance’ funding round. This programme invested almost £18 million in 10 projects that supported over 7300 children and young people through diversion programmes, providing alternatives to arrest, conviction, and custody.

A summary of UK-developed tertiary prevention projects is available in Appendix A. In the sections that follow, we provide a summary of the main projects and their rationale under each of these categories. Where the findings of impact evaluations have been published, we summarise these, with the projects in aggregate providing the dataset on which the rest of the article is based. The article focuses on interventions aimed at young people already involved in the criminal justice system. Whilst many interventions focus solely on those interventions, some also include children at high risk of being involved in the criminal justice system (secondary prevention). We include both examples in our review, but in the appendices and in the text, we try to make clear when an intervention also includes a secondary prevention focus.

#### 3.1.1. Point of Arrest Diversion

Point of arrest diversion has been a key part of YEF’s investment in tertiary prevention. Point of arrest diversion allows young people to avoid a criminal record by completing a diversionary intervention. This intervention can take many forms, as illustrated by the following projects.

Restorative justice is an approach with an established international evidence base (Beckman et al., 2024; Hobson et al., 2022), but a comparative paucity of UK evidence concerning children and young people. Typically a voluntary process, restorative justice supports the person who committed an offence and those affected in communicating and seeking to repair the harm caused. The REMEDI restorative justice project (Bandyopadhyay et al., 2024) works with children and young people who have displayed violent behaviours and/or have committed violent crimes. Practitioners facilitate restorative justice interventions between the young person and any identified victims, alongside providing intensive mentoring support for the young person and additional support for their families. YEF funded an RCT (efficacy study) looking at the impact on offending behaviour. The evaluation results will be available later in 2026.

The Salford Foundation’s STEER Programme (Boxford et al., 2023, 2026) is implemented by youth workers (mentors), who provide four weeks of initial interactions and assessment, followed by 24 weeks of weekly, face-to-face, one-to-one, one-hour mentoring sessions, and one hour per week of wraparound casework. Whilst international evidence suggests mentoring is associated with a moderate impact on reducing violent crime (Youth Endowment Fund, 2022), the STEER evaluation fills an evidence gap in what works in an English and Welsh context. YEF funded an RCT (efficacy study) examining the impact on offending behaviour; young people diverted from the criminal justice system were randomised to receive STEER or services as usual. Children who received STEER were slightly more likely to report offending behaviour post-intervention than those who did not, though this difference was not statistically significant. Importantly, STEER may not have been delivered at the intended intensity. Children were recorded as having received, on average, 14 one-to-one mentoring sessions rather than the intended 24, and so we recommended that attendance and engagement be carefully monitored and that strategies be implemented to reduce barriers to attendance.

REFRAME is a diversion programme for 10–17-year-olds in police custody who have been found in possession of class B or C controlled drugs, that aims to reduce substance use and offending (Coulton et al., 2024a, 2024b). It provides two sessions, approximately 45 minutes each, delivered by youth substance misuse workers. YEF funded an RCT (efficacy study) examining the impact on offending behaviour; young people diverted from the criminal justice system were randomised to receive REFRAME or services as usual. Children who were diverted to REFRAME were more likely to commit offences than children who were diverted but did not receive the programme, though this finding was not statistically significant (Coulton et al., 2026). Secondary outcomes were mixed, with the comparison group reporting better outcomes with regard to emotional regulation, well-being, quality of life and expectancies about the negative effects of substance use; but faring poorer on prosocial and conduct behaviour and motivation to change, relative to the REFRAME group. Given the effectiveness of diversion programmes in systematic review evidence (Keenan et al., 2025), it is important to reflect on why REFRAME showed no consistent benefit over usual services. It is worth noting that the study population had a relatively low level of prior offending and were relatively infrequent substance users. Where baseline levels of offending are low (e.g., a floor effect), it is inherently more challenging to detect measurable changes in outcomes (Šimkovic & Träuble, 2019). The authors therefore hypothesise that substance use interventions may be better targeted at young people who screen positive for a substance use disorder (Coulton et al., 2026).

#### 3.1.2. Hospital-Based A&E Navigators and Custody Suit Diversion

Hospital navigator programmes have an emerging and rapidly expanding evidence base, including multiple recent evaluations and reviews (e.g., Godwin et al., 2023; Nofi et al., 2023; C. R. Smith et al., 2026). A Cochrane review of navigator programmes for chronic illness found insufficient evidence for their use among children and adolescents with chronic diseases (Lalji et al., 2024), which adds to the case for evaluating navigator programmes for young people who have been victims of violence. YEF has funded three hospital-based A&E navigator projects (one quasi-experimental evaluation, one feasibility study and an implementation and process evaluation) and three custody suit diversion projects (two pilot studies and one efficacy study).

These interventions are typically underpinned by two key premises. The first is that diversion can act as a protective factor, reducing the likelihood of further violence or offending behaviours. This could be because of the impact of the diversionary activities themselves, such as mentoring, therapy and restorative justice. It could also be that the very act of diversion itself has a protective factor by reducing the negative impact that being processed through the criminal justice system may have on young people (Adler et al., 2016). For example, The Edinburgh Study in Youth Transitions and Crime (McAra & McVie, 2007) found that those brought to court were twice as likely to commit another offence within 12 months than a matched sample not brought to court. The second underpinning idea is that being arrested (Lawson & Flocke, 2009) or being in hospital after an injury (Wortley & Hagell, 2021) constitutes a reachable and teachable moment that can serve to strengthen the effect of behaviour change interventions. The methodological challenges involved in these types of projects are described in Section 4.

#### 3.1.3. Mentoring for At-Risk and High-Risk Young People

Mentoring is another widely implemented approach across many of YEF’s sectors. Mentoring schemes vary in terms of programme model, target groups, intervention duration, intervention location, and mentor characteristics (DeWit et al., 2016; Youth Endowment Fund, 2022). Overall, evidence demonstrates that mentoring is likely to have a moderate impact on reducing violence and reducing reoffending (Lakshminarayanan et al., 2022; Raposa et al., 2019; Tolan et al., 2013) though there is a paucity of research in the UK context.

The mentoring schemes funded by YEF vary in terms of programme model duration, target sample of young people, and mentor characteristics. At its core, mentoring involves a sustained interaction between the mentor (trusted adult) and the mentee (young person) with the aim of improving behavioural, social and emotional competencies, as well as longer-term academic, substance misuse, crime and violence outcomes. Many of these interventions focus on both secondary and tertiary prevention. Two of the mentoring projects funded by YEF include additional components, such as music as a hook (United Borders, Bandyopadhyay, 2023; Audio Active, Lord et al., 2024). Two other projects are focused specifically on young people already involved in the criminal justice system, pairing them with mentors who have lived experience of the challenges facing young people (Spark2Life Meaningful, Barnard, 2024; SOS+ programme Magić, 2023). Others are the Welsh Cerridewen project, which blends mentoring with case management (Irani et al., 2024); and the EXODUS project, which focuses on mentoring implemented by mentors trained in restorative practice (Stanford et al., 2024). All these evaluations are RCTs (efficacy studies), either looking at the impact on offending behaviour or using the Strengths and Difficulties Questionnaire (Goodman, 1997).

#### 3.1.4. Therapeutic Interventions

Therapeutic interventions funded by YEF have focused either on young people at risk of extra-familial harm (Your Choice, Rasul et al., 2021), or on young people with complex needs who are giving cause for professional concern, including posing a risk of harm to others or potentially being involved in the youth justice system (Mentalization-Based Therapy for Conduct Disorder, Edbrooke-Childs et al., 2025).

The Your Choice evaluation (Cattan et al., 2026) is possibly the largest impact evaluation ever carried out within the UK’s youth sector, an RCT (efficacy study) involving all 32 London boroughs and 1402 young people. Your Choice demonstrated a small impact on reducing self-reported severe conduct problems amongst children. After the programme, children assigned to Your Choice practitioners were very slightly less likely to report severe conduct problems than their counterparts who were not assigned to Your Choice practitioners, though this difference was not statistically significant. As with the STEER project mentioned earlier, Your Choice implementation struggled with fidelity issues: in the Your Choice group, fewer than 1% of children received 36 or more sessions (the expected number). Further, 16% of children received no sessions, 47% received 1–9 sessions, and 26% received 10–19 sessions. Consequently, YEF concluded that, due to fidelity issues and methodological challenges related to attrition, any future consideration of the programme would require material changes to the delivery model rather than minor adjustments.

### 3.2. Adapting International Evidence-Based Interventions for Evaluation in the UK

Reviews of prevention interventions (Asmussen et al., 2018; Wilkinson et al., 2024) tend to show a mix of ‘home-grown’ interventions developed in the UK, alongside evidence-based interventions developed in other jurisdictions, notably the US. Adapting US-developed prevention interventions for the UK context has a long history, with examples including Family Nurse Partnership (Robling et al., 2016), Incredible Years (Hutchings, 2012), the Good Behaviour Game (Humphrey et al., 2022) and Multisystemic Therapy (Fonagy et al., 2020). There is also a significant body of literature in prevention and implementation science on how best to adapt interventions to new contexts (Copeland et al., 2021; G. Moore et al., 2021; Movsisyan et al., 2021; Wiltsey Stirman et al., 2019).

Three insights motivated YEF’s efforts to adapt interventions to the UK context, drawing on lessons from previous adaptations. First, given the urgency of tackling youth crime, taking empirically supported interventions and adapting them to different contexts and populations is a crucial activity, given that the cost of developing new interventions from scratch is high (Kumpfer et al., 2002). Second, it is clear that ‘context matters’ and even the most carefully designed interventions will not be successful in every context (Leviton & Trujillo, 2017; Wandersman et al., 2000). This means it is necessary to evaluate and adapt interventions with an international evidence base. Third, great care must be taken to select interventions that match the needs and goals of local populations and are evidence-based to meet those needs (Wandersman et al., 2000). Taken together, these insights shaped YEF’s strategy of prioritising adaptation alongside rigorous evaluation, rather than assuming that interventions with strong international evidence transfer directly to UK contexts.

YEF’s adaptations and evaluations of evidence-based interventions have resulted from a mixture of the commitment of others within the field in making applications for funding, and the strategic efforts of YEF and its partners, usually led by the Ending Youth Violence Lab (Ending Youth Violence Lab, n.d.). Two of our earliest efforts were adaptations of Becoming a Man (Green et al., 2023b, 2024) and Functional Family Therapy-Gangs (Humayun et al., 2023a). See Appendix B for a list of all the projects.

YEF was interested in the applicability of Becoming a Man (BAM) to the UK context due to the intervention’s strong prior evidence from the US on reducing crime (Heller et al., 2013, 2017). BAM is a school-based intervention aimed at young men who are at risk of poor academic achievement and most likely to come into contact with the criminal justice system. It is delivered by counsellors to adolescents, both individually and in groups, for two years (Foundations, 2025). Significant work went into adapting the intervention to the UK context, as described elsewhere (Green et al., 2023a). A feasibility study (Green et al., 2023b) and a pilot study (Green et al., 2024) found the quality of delivery to be generally successful, and provided insight into how best to evaluate the intervention in a future RCT, which YEF is currently in the process of designing with partners.

The Functional Family Therapy-Extra Familial Harm intervention (formerly Functional Family Therapy-Gangs) also fills a crucial gap in provision. Child criminal exploitation by county lines drug networks is a serious safeguarding issue in the UK (Brewster et al., 2023; Olver & Cockbain, 2021), with a paucity of effective interventions hampering efforts to tackle it systematically. This is what made investment in the UK adaptation of Functional Family Therapy-Gangs (FFT-G) so promising. The adaptation of FFT-G builds on the UK experience of ‘standard’ FFT (Humayun et al., 2017), whilst focusing specifically on the risk factors associated with gang involvement, which are targeted with skills training with the family. Whilst the pilot trial was impacted by the COVID-19 pandemic (Lange et al., 2023), it found sufficient evidence for the feasibility of delivery and the acceptability of running an RCT. Therefore, YEF has made an investment in a definitive trial, which is ongoing (Humayun et al., 2023b) and will report in 2027.

Focused Deterrence (FD) is a major programme of work in tertiary prevention. This three-pronged intervention combines clear *deterrence* messaging, that is, communication of the consequences of violence and swift and certain enforcement if violence occurs, with the offer of *support* that creates opportunities for desistance or positive routes away from violence. The local *community* is also engaged, communicating that they want violence to stop and for those involved to be safe, supported and reintegrated into the community (Gaffney et al., 2021b). The international evidence base on FD (Braga et al., 2019) suggests a high impact on reducing violent crime (Gaffney et al., 2021b).

YEF and the UK Home Office made a multi-year investment of £7 million in an RCT (YEF, 2022) involving nearly 3000 participants across five sites; of these, around 40% were under 18 years old at the point of randomisation (Simanovic et al., 2024). Early-stage trial findings showed that implementation was moderately successful (I. Brennan et al., 2024). There were some fidelity issues that required monitoring, particularly concerning the combined delivery of deterrence messaging with a support offer, though these were rectified in later stages of the trial (I. Brennan et al., 2026). Overall, the trial is on track to be a well-powered RCT with minimal attrition, and results from the impact evaluation will be published in 2028.

More recent adaptations have focused on the evidence-based parenting interventions GenerationPMTO (GenPMTO) (Cai et al., 2022; Forgatch & Kjøbli, 2016) and Multidimensional Family Therapy (MDFT) (Filges et al., 2015), both of which have been supported by the Ending Youth Violence Lab. GenPMTO is aimed at caregivers of 8–14-year-olds identified to have risk factors associated with involvement in violence, whereas MDFT is a multicomponent therapeutic intervention for families with a 13–18-year-old with an identified behavioural or substance misuse problem (Cai et al., 2022; Forgatch & Kjøbli, 2016). For GenPMTO, YEF contributed funding to an adaptation and feasibility study (Rahal et al., 2023) and pilot RCT (Martin et al., 2025), and will decide in 2026 whether to proceed to an efficacy study.

For MDFT, given links between substance use and later offending (Ullman et al., 2024), YEF invested in an RCT targeted at 400 young people who use alcohol and/or drugs and who have offended or are at high risk of offending (Coulton et al., 2025), with findings due in 2029.

### 3.3. ‘Multi-Site Trials’: A Group Approach to Carrying out Randomised Controlled Trials

We know from previous evaluation efforts that underpowered trials that struggle to recruit the required number of participants are the norm rather than the exception (Epstein & Klerman, 2012). This created an imperative for YEF to find additional approaches to conducting RCTs that we know require large numbers of children to participate. Building on work that one of the authors commissioned whilst in the UK Cabinet Office (Walshe et al., 2016), YEF extended the standard trial concept of a ‘multi-site trial’ to address three evaluation challenges faced in the sectors within which YEF operates. First, whilst evaluating well-designed manualised interventions is an important part of YEF’s strategy, much of the work in the youth sector is delivered by small local organisations and comprises a range of non-manualised, flexible approaches (Rowland et al., 2024). Second, RCTs can be out of reach for many grassroots organisations because they are, by definition, often small and locally focused, and do not reach the hundreds, and potentially thousands, of children required for most trials. Third, the challenge of scale could undermine the equitable distribution of YEF’s funding. We know from YEF’s early funding rounds that delivery organisations led by people from minoritised backgrounds tend to be smaller in scale, so focusing only on large trials with a single delivery organisation would likely crowd out these minority-led organisations.

The experience of YEF’s early funding rounds suggested that whilst there is great diversity in approaches to crime and violence reduction interventions, there are some key activities, such as mentoring and sports-based approaches, that are common across many different delivery partner organisations (DPOs). This creates the possibility of working with multiple DPOs within a single RCT. We tested the multi-site trial approach through a feasibility study (Hall et al., 2023) and the impact study (RCT) of mentoring (Rowland et al., 2024). Taken together, these studies demonstrated the feasibility of using a multi-site trial approach to test the impact of interventions delivered by small organisations. Key components of the project’s success were the development of a shared practice model to ensure a reasonable level of consistency in delivery across DPOs and the extensive support evaluators provided to the DPOs to engage in the evaluation. With these tools in place, DPOs showed great willingness and flexibility to support trial delivery.

We recognise the potential tension between developing a shared practice model and the aim of including small organisations with ‘non-manualised’ approaches.^1^ This directly reflects a broader tension noted in the literature between standardisation for evaluation and flexibility for real-world delivery (e.g., Castro et al., 2004). The way this tension was handled in the mentoring multi-site trial was to agree on ‘core’ and ‘flexible’ components of the mentoring (Lewis et al., 2021). The shared practice model was deemed acceptable to mentors, which may have been because it aligned sufficiently closely to their existing practice, suggesting that the balancing of core and flexible components was successful (Rowland et al., 2024).

Whilst this initial work demonstrated ‘proof of concept’ in the context of secondary prevention, YEF has continued work on multi-site trials, including in tertiary prevention. The Toward Sport evaluation (Spyropoulas et al., 2025) involves over 3000 young people aged 10 to 17 and examines the impact of a sports-based intervention on violent and non-violent offending recorded in the Police National Computer (in the context of both secondary and tertiary prevention). The Toward Sport intervention is a 24-week programme, with sports-based activities led by a paid coach. There are three main ways in which sports-based activities could contribute to crime and violence prevention. First, sport could support positive social and cognitive development. Second, sport could play a role in direct prevention by reducing the time children are exposed to negative influences and allowing them to take risks in a safe environment. Third, sport could act as a ‘hook’ to engage children in other helpful interventions that could drive positive social and cognitive development (Gaffney et al., 2021c). YEF is also actively exploring other multi-site trial opportunities, including martial arts approaches and therapeutic approaches. See Appendix C for a list of all the projects involving multi-site trials.

### 3.4. Embedding a Focus on Race Equity in Tertiary Prevention: A Case Study of a Multi-Site Trial

Racial and ethnic disproportionality in criminal justice is a well-established and persistent feature of the UK context and in many other jurisdictions, with extensive evidence documenting unequal outcomes across multiple stages of the system (Gwata et al., 2024; Lammy, 2017; Ministry of Justice, 2024; Parmar et al., 2020). For example, research from the Youth Justice Board (Brodie et al., 2025) found that minoritised children, especially Black and Mixed Heritage children, were often subjected to over-policing. YEF’s racial disproportionality report (Youth Endowment Fund, 2025e) supports this, finding that Black children were 64% more likely to be arrested, 84% more likely to be convicted or cautioned and 300% more likely to be in custody, compared to their share of the population. These two studies are part of a significant international literature documenting the disproportionality in the criminal justice system (Lehmann & Meldrum, 2024; Oglesby-Neal & Peterson, 2021; Phillips & Bowling, 2017; Sampson & Neil, 2024).

Fortunately, efforts to ground race equity in funding and research programmes benefit from a rich body of established scholarship and policy work on race and inequality, which emphasises the need to explicitly address structural inequities (El-Enany & Bruce-Jones, 2015; Goodrum et al., 2024; Jones, 2000; Murry et al., 2024; Phillips, 2011; Walcott & Nightingale, 2025). This shift has important implications for intervention design and evaluation, as programmes developed and tested in majority populations may not demonstrate equivalent impacts across racially minoritised groups, particularly where underlying mechanisms of risk are shaped by structural inequalities (e.g., Bernal & Adames, 2017; Castro et al., 2004).

Drawing on these perspectives, and recognising that experiences of violence and interventions are shaped by intersecting forms of disadvantage (e.g., Peguero & Popp, 2012; L. Roberts et al., 2018), YEF’s approach to embedding race equity can be understood as part of a much broader shift towards equitable models of evidence generation, synthesis and policy development (Diderichsen et al., 2022; Jull et al., 2017; Krieger, 2021; Mbuagbaw et al., 2017; Petkovic et al., 2020).

Nevertheless, efforts to embed race equity within intervention design and funding face several challenges. There is a risk that programmes that are not explicitly designed with cultural and contextual relevance may fail to engage the populations most affected or may inadvertently reproduce existing inequalities. This means that a concerted effort is required to ensure that all children have a positive experience throughout their involvement in the programme and the evaluation.

Minoritised-led grassroots organisations typically exist because the client groups they serve are not adequately served by mainstream organisations, yet these organisations face significant barriers to and discrimination from traditional funding structures (Butt, 2001; Manful & Willis, 2024; Tilki et al., 2015). In recent years, many initiatives across the funding system have tried to address this issue (Uwadiae et al., 2026). YEF has tried to build on these initiatives by providing minoritised-led opportunities to take part in robust impact evaluation, as demonstrated in the Moves Different boxing trial to reduce offending (McBride et al., 2025). Following the principles laid out in the previous section, the project was set up as a multi-site trial recruiting participants in both secondary and tertiary prevention contexts. In practice, this means a single umbrella organisation worked with 40 boxing clubs or delivery partner organisations (DPOs). To redress the underfunding of organisations led by people from minoritised backgrounds, 60% of the DPOs are led by racially minoritised leaders. Furthermore, at least 30% of the 3600 young people in the trial will be from minority ethnic backgrounds. This example illustrates how embedding race equity within funding models requires not only proportional inclusion but also active restructuring of delivery partnerships and access to evaluation infrastructure. This is an initial step to redressing one of the ways in which systematic barriers manifest in funding structures oriented towards impact evaluation.

Unfortunately, systemic barriers are not limited to the criminal justice system or to intervention funding but are also present within the research process itself. People from minority ethnic backgrounds are systematically underrepresented in trials (Hussain-Gambles et al., 2004; Pardhan et al., 2025; Redwood & Gill, 2013); they also tend to be underrepresented in the research professions (S. O. Roberts et al., 2020; The Young Foundation, 2021). Inspired by existing guidelines from NIHR (2025) and Child Trends (Andrews et al., 2019), YEF supports partners in embedding race equity considerations throughout the design, execution, reporting and dissemination of research. For example, during the setup of the Moves Different trial, the delivery and evaluation teams collaborated with a YEF Race Equity Associate (REA). This initiative was set up in 2023 to strengthen our research and evaluation work by partnering with consultants with practical experience in embedding a focus on race equity within the research process. In the Moves Different trial, the REA attended and contributed to the co-design of the evaluation and provided feedback on the intervention’s shared practice model and the evaluation design and protocol. Later in the research process, the REA will support the qualitative analysis to ensure the research team’s positionality is considered, and that an equitable approach to analysis is fostered throughout.

Despite the success of the REA initiative, we recognised that we must go further in ensuring that data collection is racially equitable and that all children and young people have a positive experience of the research process. This led to YEF establishing the Race Equity Data Collection Panel (Youth Endowment Fund, 2025d), aiming to improve the cultural competency of the researchers we work with. The Moves Different trial includes an organisation from the Race Equity Data Collection Panel, ClearView Research, which specialises in amplifying the voices of underrepresented, minority ethnic and seldom-heard communities. ClearView’s involvement led to two major initiatives to improve the research process. The first initiative was the introduction of ‘youth participatory panels’ to understand young people’s views on the intervention and on taking part in the evaluation. This initiative can be understood as part of wider efforts to improve the voice of children and young people in the design of research aiming to improve their lives (Lipton et al., 2025; Wyatt et al., 2024). The second initiative was the introduction of ‘trusted guides’, early-stage researchers with lived experience, to improve engagement and retention of young people in control groups, among whom lower engagement rates are commonly observed (Wolff, 2000). Given the benefits of peer researchers in other contexts, we are optimistic about the potential of this initiative to improve the robustness of the trial. YEF will continue to use its position to question and challenge, facilitated by annual race equity goals (Youth Endowment Fund, 2025f) and the publication of an annual race equity report (Youth Endowment Fund, 2025g).

## 4. The Design of High-Quality Evaluations of Tertiary Prevention

### 4.1. Required Scale for Evaluation

Crime is, generally speaking, a rare event (Prieto Curiel et al., 2018), with around 150 incidents per 1000 people in England and Wales (9.3 million incidents with a population of just over 60 million) in the year ending June 2025. Violence is even rarer, with around 18 incidents per 1000 people over the same period (1.1 million incidents), or affecting just under 2% of the population annually (ONS, 2025). Therefore, in order for evaluations of crime and violence reduction interventions to generate robust evidence of impact (or lack thereof), they need to be at a considerable scale and adequately powered to detect meaningful intervention effects.

To further complicate matters, children and young people who are at high risk of involvement or have been involved in serious violence or organised crime, tend to desist naturally over time (i.e., become less likely to commit crime and violence as they grow older), leaving a relatively small group who go on to be long-term perpetrators of crime and violence. This implies that tertiary samples of children and young people may have lower reoffending rates than expected, so even larger samples may be required to adequately power evaluations of interventions targeted at tertiary samples.

There are several ways to think about scale and the corresponding trade-offs. These can be done from the outset when designing and setting up the evaluation, including when running power calculations or simulations:

- Sample size—sample size can be increased by:
  ○ Recruiting more participants. This can be done by ensuring recruitment and referral pathways are well set up, with evidence of good throughput, and/or by using small-batch randomisation or rolling recruitment (described further below).
  ○ Engaging more delivery sites (see our MST examples above, e.g., Toward Sport), delivery partner organisations (e.g., Your Choice) or settings. It is worth noting that the more delivery partners involved—each with their own established practices and local contexts—the greater the challenge of developing a shared practice model and maintaining fidelity to the intervention.
- Target population
  ○ Whilst it is sensible to focus on tertiary cohorts, among whom we would expect larger and more meaningful changes in the outcome of interest, this cohort can be rare or difficult to recruit, depending on the delivery setting.
  ○ For some interventions and settings, it may be worth considering a blend of secondary and tertiary cohorts to ensure adequate numbers for power; however, this may exacerbate the scale issue if the primary outcome of interest is relatively rare in the target population.
- Outcome measure
  ○ Employing a primary outcome measure that is more sensitive to changes in criminal or violent behaviour and delinquency outcomes (i.e., greater variation in the outcome), with minimal floor/ceiling effects (e.g., avoiding measures with a very large proportion of zeros), can reduce the minimum sample required for an adequately powered and robust evaluation.

The following sections discuss lessons we have learned in ensuring and maintaining an appropriate scale across our evaluations.

### 4.2. Consenting Young People to Trials

When commissioning the evaluation of any intervention, it is critical to ensure all legal and ethical requirements, including any necessary consent procedures, are followed. However, in the context of tertiary interventions, obtaining informed consent can pose challenges. Many eligible participants may be in custody suites, known to be frequently ‘missing’ from home or care, or potentially unresponsive when contacted. Even if the intervention delivery team get into close contact with them, getting them to consent to a needs-based, personalised, one-to-one intervention and agreeing to the requirements of the evaluation (e.g., completing questionnaires and allowing access to their personal data and offending records) would be challenging. Further complexities may arise when there are underlying trust issues with the system and service providers, particularly in the case of minority ethnic young people and their parents or carers.

The Redthread Youth Violence Intervention Programme is an A&E navigator service for young people who present to hospital emergency departments following a violent incident, or an incident putting them at risk of involvement in violence. A YEF-funded pilot evaluation of this programme aimed to compare data from all referred 10–17-year-olds across five hospitals, with those in the same hospitals who did not receive the programme, and required eligible participants to consent to join the study and provide personal and outcome data. However, many young people were in pain, distressed or dealing with trauma and safety concerns, making it inappropriate or difficult for youth workers to approach them about the evaluation and to get their consent. Ultimately, only one child (out of an expected pilot sample of 150) signed up, and the evaluation design had to be pivoted to a retrospective quasi-experimental evaluation (Montgomery & Chandan, 2022).

Similar issues were observed in YEF evaluations aimed at recruiting young people already in custody settings. Across three pilot evaluations of pre-court diversionary programmes (DIVERT, Divert Plus and Brief Solution Focused Therapy), obtaining informed consent from young people in custody proved extremely difficult, and primary data collection was often incomplete. Considering the low rate of consent with this population, a decision was made in the Brief Solution Focused Therapy evaluation (Thompson & Playle, 2025), to recruit additional custody suites so as to be able to approach a larger number of young people in custody—note that this naturally requires more resources, and may not be feasible for some contexts and funders. The young people approached are also those already engaged with services, increasing the likelihood that they will consent to the evaluation.

When primary data are being collected directly from participants, informed consent is a foundational part of research practice. In such evaluations, ethical review boards would expect evaluators to inform participants (or parents or carers) that their data will be processed, by whom and for what purpose; and to obtain their specific consent to being randomised, to receiving the intervention and to their data being processed for the evaluation. However, there is no requirement for evaluators to rely on consent to *process* personal data for research purposes, even if there is a separate legal or ethical obligation to obtain consent from participants providing data for the research. We therefore recommend that, insofar as possible, instead of using consent as the legal basis for processing personal data, evaluators look to use public task or legitimate interests, and rely on the research condition for processing criminal offence and other special category data (Information Officer’s Office, 2025; Youth Endowment Fund, 2023).

One way to avoid the burden or stress that could result from trying to obtain informed consent in custody or hospital settings is to rely on administrative data already collected by local police forces, schools and hospitals (see Ellison & Cook, 2025 for a guidance to administrative UK datasets relevant to our evaluations), instead of data collected directly from participants, such as via interviews, surveys or questionnaires. It is crucial to note that these require appropriate Data Sharing Agreements and potentially data management platforms in place, both of which can lead to significant delays in the evaluation timeline—this was a substantial consideration in a feasibility study of a multi-site hospital navigator service (Sutherland et al., 2023). The above considerations and further mitigations are summarised in Table 1.

More generally, the evaluation design and pre-specification period should involve strict prioritisation of the data types and amount that are truly vital to the evaluation, consideration of all alternative data sources, and the building and maintenance of safeguards for the responsible and ethical use of personal data. Naturally, all of these must be balanced against the intended rigour, robustness, power and representativeness of the evaluation.

The Focused Deterrence trial provides a suitable case study. With nearly 3000 participants, the RCT is powered to detect impacts as low as a 20% relative reduction in the number of violence against the person offences (the primary outcome for the impact evaluation). Personal data to which evaluators have access for purposes of the evaluation (e.g., retrospectively assessing eligibility, analysing results) are minimal and organised via unique reference numbers rather than any identifying information (e.g., names, dates of birth, full addresses); these are also shared, stored and protected via secure infrastructures. Data are obtained from the following sources and processed under the legitimate interests provision (I. Brennan et al., 2024):

- Police records. The primary and secondary outcomes of interest (across both intervention and control groups) are from police administrative data, for which Data Sharing Agreements between evaluators and forces have been set up at the outset of the evaluation. These include criminal records and ethnicity information, without any other identifying information, and are accessed only at pre-specified intervals and at the end of the follow-up period.
- Intervention process data. Participants who are randomised to receive the intervention and who engage with the programme consent to intervention delivery teams collecting and pseudonymising their data (linked to police records via a unique identifier) for sharing with the evaluation team.
- Contact information for participation. Participants in the intervention group who are recruited to complete qualitative interviews (for the implementation and process evaluation) are asked to consent to sharing their contact details with the evaluators. However, care is taken to collect only the minimum amount of personal information necessary to contact participants for interviews or for observations of intervention delivery.
- Qualitative interview data. Participants in the intervention group who agree to participate in qualitative interviews are asked to provide informed consent or parental/guardian assent (for young people). A clear flipside is that participants in the control group would not be recruited for any qualitative interviews.

## 5. Risks, Challenges and Lessons Learned in Recruiting and Retaining Young People in Tertiary Prevention Evaluations

### Core Risks and Corresponding Challenges and Mitigations in Recruitment and Retention

We structure this section around four recurrent risks, outlining the associated challenges and the mitigation strategies that have emerged across the portfolio. Recruitment and retention into trials is challenging, with a large body of literature documenting facilitators and barriers to the success of robust trials (e.g., Axford et al., 2023; Berry et al., 2023; Ramakrishnan et al., 2022). Recruitment and retention have consistently been among the highest risks to evidence quality in YEF’s tertiary prevention evaluations. This is not a function of young people being “hard to reach”, but of trials being conducted in volatile systems involving young people who tend to show high mobility, inconsistent contact with services, and frequent transitions between custody, care and community settings, thereby challenging conventional evaluation timelines. Across our portfolio of almost 60 active evaluations, we have learned that recruitment and retention challenges are often early indicators of deeper misalignment between trial design, delivery realities and system incentives. This section focuses on those lessons, drawing on examples from YEF-funded trials.

**Risk 1: Fragile referral pipelines and gatekeeper dependence**

A recurring recruitment risk has been the reliance on complex, multi-agency referral systems to access tertiary cohorts. In custody-suite diversionary programmes, hospital-based interventions and high-risk mentoring programmes, recruitment depends on police, local authorities, health services, schools and voluntary sector partners acting in concert. In practice, referral pipelines have often taken longer than anticipated to mature or have functioned intermittently due to staff turnover, competing operational priorities and data-sharing frictions. In several large local-authority-led trials, early recruitment shortfalls were not driven by a lack of eligible young people, but by frontline staff being unclear about eligibility criteria, uncertain about explaining trial processes, or hesitant about the random allocation of participants into intervention and control groups.

**Mitigation: Distributed ownership and flexible recruitment models**

The most robust recruitment approaches have been those that avoided reliance on single gatekeepers and instead distributed responsibility across teams and agencies. Trials that made recruitment roles explicit, monitored referral activity, and built redundancy into pipelines were more resilient. Flexible recruitment models, such as rolling recruitment, cohort-based intake or small-batch randomisation, have also been particularly important in tertiary prevention. Although these approaches complicate timelines and power calculations, they better align evaluation processes with reachable and teachable moments in young people’s lives. Several trials demonstrated that long delays between referral, randomisation and the start of intervention materially reduced engagement, even where consent had already been secured.

**Risk 2: Uneven acceptance of randomisation**

Randomisation has been a recurrent source of recruitment risk, reflecting uneven familiarity and comfort with experimental methods across partner organisations. Where randomisation was introduced using technical or evaluative language, it sometimes slowed referrals—not because of principled opposition to evaluation, but due to concerns about fairness, professional judgement and safeguarding. Recruitment risk was highest where acceptance of randomisation was assumed rather than actively maintained.

**Mitigation: Ongoing communication and operational responsiveness**

Effective mitigation has required treating acceptance of randomisation as an ongoing process rather than a one-off design decision. Trials that invested in repeated, practical conversations with frontline staff, framing randomisation in terms of fairness, learning and system improvement, were better able to sustain recruitment momentum. More broadly, YEF has learned to treat recruitment challenges as legitimate triggers for mid-course operational redesign. Whilst core design features must remain fixed, revisiting eligibility criteria, referral thresholds, and recruitment timelines has been essential in large, multi-site trials to preserve feasibility and protect evidence quality. Regular, structured monitoring of recruitment has been critical in surfacing these issues early enough to act.

**Case study: Functional Family Therapy-Extra Familial Harm (FFT-EFH)**

The FFT-EFH evaluation in local authority contexts demonstrated how an inconsistent understanding of randomisation can slow recruitment. Frontline local authority staff were initially hesitant to refer families into a trial with randomised allocation, expressing practical concerns about fairness, discretion and how families would perceive assignment to the control condition. Early referral flows lagged because randomisation was introduced in technical terms that did not resonate with practitioners’ day-to-day decision-making.

To address this, delivery partner staff, with the support of the evaluator, visited referral teams to explain the purpose and practical mechanics of the trial in plain terms, framed around fairness and learning rather than heavy reliance on research jargon. This reinforced confidence among gatekeepers, increased the rate and consistency of referrals, and helped embed a shared understanding of why randomised designs are necessary to answer “what works” questions in practice.

**Risk 3: Measurement attrition in high-mobility cohorts**

Measurement attrition has been one of the most acute and persistent threats to evidence quality in YEF’s tertiary prevention evaluations, particularly where primary outcomes rely on self-report measures. A substantial proportion of YEF-funded trials use validated self-report measures such as the Strengths and Difficulties Questionnaire (Goodman, 1997) or Self-Report Delinquency Scale (D. J. Smith & McVie, 2003) with cohorts characterised by high mobility, unstable contact details, and frequent transitions between custody, care and community settings. Even where initial recruitment has been successful, follow-up rates at mid-point and endline have often fallen well short of initial assumptions.

Importantly, measurement attrition in these contexts is not evenly distributed. Loss to follow-up is more common among young people experiencing acute instability or disengagement from services, raising the risk of systematic bias and reducing statistical power. In several evaluations, attrition emerged only after delivery was well underway, at which point options to address imbalance or recover missing data were limited.

**Mitigation: Active monitoring, evaluator capacity and data strategy**

YEF’s experience underscores the importance of treating measurement attrition as a dynamic risk that requires active management throughout delivery, rather than as a problem to be addressed retrospectively during analysis. Regular monitoring of follow-up rates from the point of randomisation, including differential attrition between trial arms, has enabled earlier identification of emerging risks and more timely corrective action. Trials with dedicated evaluator resources for follow-up, such as research assistants responsible for sustained contact via calls, texts and in-person visits, have consistently achieved higher retention than those relying solely on assumed best practices or practitioner goodwill.

In addition, YEF has increasingly emphasised the strategic use of administrative data as primary or supplementary outcome sources, where appropriate. Whilst not a panacea, using administrative data has reduced reliance on repeated participant contact and mitigated attrition-related bias in several tertiary prevention trials.

**Case study: Your Choice (London VRU)**

The Your Choice efficacy trial is a clear example of measurement attrition risks in tertiary contexts. Although recruitment enrolled over 1600 children, 44% of participants were excluded from the primary outcome analysis because they did not complete the required measures at follow-up. This substantially reduced the trial’s evidence quality and constrained confidence in its conclusions.

Throughout delivery, the evaluation and delivery teams closely monitored attrition, using multiple contact methods and structured tracking processes to stay in touch with participants as the trial progressed. Whilst attrition remained high, it was reduced from 60% in the final year of the trial. This continuous monitoring enabled the team to identify dropout patterns early and refine follow-up strategies for mid-trial engagement, highlighting the importance of active, adaptive management rather than relying solely on initial recruitment.

**Risk 4: Intervention fatigue and cumulative participant burden**

Linked to measurement attrition, intervention fatigue has emerged as a significant driver of disengagement in tertiary prevention evaluations. Young people in these trials are often simultaneously involved with multiple statutory and voluntary services, court processes, education transitions or placement changes. Against this backdrop, evaluations that layer extensive outcome batteries, repeated follow-up points, or duplicative data collection onto already intensive interventions risk overburdening participants.

Intervention fatigue has been particularly evident where data collection is practitioner-led without sufficient evaluator support, and among control group participants or those who disengage early from the intervention. In these cases, attrition reflects not only instability but also rational disengagement from processes perceived as burdensome or low value.

**Mitigation: Burden reduction, prioritisation and incentives**

The most effective mitigations for intervention fatigue have been design-led and pragmatic. Reducing participant burden by prioritising primary outcomes, removing non-essential repeated measures, and simplifying data collection instruments has materially improved retention in several trials without compromising core research questions. Aligning data collection more closely with existing service touchpoints has also reduced duplication and participant frustration.

Incentives, both financial and non-financial, have played a complementary role when applied transparently and consistently. These have been most effective when offered to both intervention and control participants or institutions, reinforcing perceptions of fairness and recognising the time and effort involved in participation. YEF has increasingly supported mid-point reallocation of budget towards follow-up and retention activities where there is a strong rationale that modest additional investment can substantially reduce fatigue-related attrition and protect overall evidence quality.

**Case study: Portfolio-wide retention responsiveness**

YEF has established a contingency pot of ringfenced funding and a streamlined variation process that allows projects experiencing high attrition to access rapid, targeted funding. This can be used, for example, to enhance evaluator follow-up capacity or to introduce incentives, without extensive bureaucratic delay. This responsive mechanism helps teams act quickly at ‘pressure points’ in delivery to reduce burden-related attrition, rather than waiting for analysis to reveal lost data, long after participants have disengaged.

**Conclusion** **s**

Overall, YEF’s experience suggests that recruitment and retention challenges in tertiary prevention are not aberrations to be managed away, but structural features of the systems in which these interventions operate. Producing high-quality evidence in this context requires not only rigorous design but ongoing, adaptive stewardship of trials as delivery realities and system conditions evolve.

## 6. Discussion

This article has reflected on YEF’s experience of commissioning and delivering large-scale evaluations of tertiary prevention interventions for children and young people already involved in, or at high risk of, serious violence. The overview of YEF’s tertiary prevention portfolio illustrates both the breadth of approaches being tested—spanning diversion at points of arrest and hospital contact, mentoring and therapeutic provision, and the adaptation of internationally evidenced interventions—and the strategic intent behind this work. Collectively, these investments represent a large-scale effort to strengthen the UK evidence base on tertiary prevention, in a field historically characterised by limited experimental evidence and high levels of implementation uncertainty.

A key insight from this work is the central importance of scale and statistical power. Evaluating impacts on crime and violence requires large samples, long follow-up periods and careful outcome selection, particularly given the rarity of serious offending and the tendency for natural desistance among high-risk cohorts. YEF’s response has been to prioritise well-powered randomised controlled trials and to develop innovative delivery and evaluation models, including multi-site trials that aggregate provision across multiple organisations and local systems. Whilst these approaches introduce additional complexity, particularly in relation to shared practice models and fidelity, they have proved essential for producing credible, decision-relevant evidence and for enabling smaller, community-based organisations to participate in rigorous evaluation.

The article also highlights the distinctive ethical and practical challenges of obtaining consent from young people for trials in tertiary prevention contexts. Settings such as custody suites and emergency departments are often characterised by crisis, trauma and acute safeguarding concerns, limiting the feasibility of conventional consent processes for primary data collection. YEF’s experience underscores the importance of early, realistic design decisions about data sources, including the strategic use of administrative data, such as police-recorded crime. These lessons have informed YEF’s wider administrative data guidance and reinforced the need to balance ethical safeguards, proportionality, and evaluative rigour when working with highly vulnerable populations.

Recruitment and retention challenges emerge not as isolated operational issues, but as structural features of tertiary prevention systems. Complex referral pathways, reliance on multiple gatekeepers, uneven familiarity with randomisation, and high levels of participant mobility consistently place pressure on trial delivery. YEF’s learning points to the value of adaptive trial stewardship, treating recruitment, retention and engagement as dynamic processes that require ongoing monitoring, flexibility and shared problem-solving among funders, evaluators and delivery partners. Pragmatic mitigations, such as reducing data collection burden, investing in follow-up capacity, and aligning recruitment processes with reachable moments, have often been critical to maintaining evidence quality.

The evaluation results of STEER and Your Choice also remind us of the importance of being clear about ‘how much’ of an intervention we expect young people to receive to achieve the desired behaviour change. In both cases, there was a significant gap between intention and reality. Prevention science has long recognised the importance of implementation fidelity (Damschroder et al., 2009; Fixsen, 2005; Gottfredson et al., 2015) and the factors that impact fidelity are relatively established in other areas of prevention science, such as social and emotional skills programmes (e.g., Durlak, 2018; Durlak & DuPre, 2008; Thierry et al., 2022) and parenting programmes (e.g., Axford et al., 2017; Bywater et al., 2019).

In contrast, youth violence prevention is at a much earlier stage of understanding the factors that influence intervention implementation and the strategies that are effective in improving implementation fidelity. The YEF Toolkit makes an important contribution to understanding the factors associated with both successful implementation and positive outcomes. In the case of mentoring (Youth Endowment Fund, 2022), it is recommended that at least six months of one-to-one sessions be offered, which provides context for why the average of 14 sessions implemented in the STEER project led to disappointing outcomes. YEF is actively exploring ways it can contribute to understanding and promoting strategies to improve implementation fidelity, such as through the delivery guidance for Focused Deterrence (Youth Endowment Fund, 2025b).

Two cross-cutting issues remind us that evaluation is not merely a technocratic exercise aimed at reducing threats to internal validity, but a deeply human endeavour that involves the cooperation and compromise of many individuals with diverse interests and priorities. In carrying out this work, we are often met with scepticism and sometimes resistance to carrying out RCTs concerning youth violence. RCTs have faced reluctance and resistance in almost every field in which they have been implemented, including in education (e.g., Oakley, 2006), international development (e.g., de Souza Leão & Eyal, 2019), medicine (e.g., Benson et al., 1991), public health (Macintyre, 2011; Macintyre & Petticrew, 2000) and social care (Dixon et al., 2014).

We have tried to balance the necessary confidence in YEF’s work with deep empathy for partners’ concerns. This has involved developing detailed material about the case for RCTs, inspired by work from Macintyre (Macintyre, 2011; Macintyre & Petticrew, 2000). The most common objection, by some margins, is that RCTs are not ethical. Our approach to this objection has been to patiently describe the robust ethical review processes that each evaluation is subject to and to interrogate the assumption that well-intentioned programmes will be beneficial, in light of ineffective or harmful approaches such as Scared Straight (Petrosino et al., 2013) and Boot Camps (Gaffney et al., 2021a). Over time, arguments that all RCTs are unethical become harder to reconcile with the dozens of trials already underway that have received ethical approval.

A further cross-cutting insight from this body of work is the centrality of race equity in the design, conduct and interpretation of tertiary prevention evaluations. Given the well-documented racial disproportionality within the youth justice system, decisions about who is evaluated, how interventions are delivered, and what data are collected are inherently equity-relevant. YEF’s experience demonstrates that improving representativeness requires action across the full evaluation lifecycle, including equitable access to funding for delivery organisations led by racially minoritised leaders, culturally competent evaluation practices, and trial designs that minimise exclusion through overly burdensome consent or data collection processes. Embedding race equity considerations into multi-site trial models, participatory design processes, and administrative data use has been essential not only for fairness but for the validity and credibility of the evidence generated.

Crucially, an important insight from YEF’s work is that generating high-quality evidence is insufficient on its own; active, sustained investment in evidence mobilisation is required to influence policy and practice. Existing research (Haynes et al., 2018) and policy analysis (OECD, 2020) show that, despite calls for policies and programmes to be evidence-based, effective connections between evidence and policy-making remain elusive (Newman et al., 2017). Fortunately, the barriers and facilitators to the use of evidence are now well understood (Oliver et al., 2014; Oliver & Cairney, 2019), with timely access to high-quality and relevant research, collaborations with policy-makers, and relationship and skill building with policy-makers amongst the most important factors in influencing the use of evidence (Oliver et al., 2014). This is why the rise of knowledge brokers (Ward et al., 2009), such as the What Works Centres, is so crucial for bridging the divide that can exist between the supply and use of evaluation evidence.

YEF’s Toolkit is a key part of the knowledge brokerage function to improve access to high-quality evidence. Findings from impact evaluations feed directly into the YEF Toolkit, which synthesises global and UK evidence on approaches to preventing serious violence, and presents accessible summaries of impact, cost, implementation and the strength of the evidence. The Toolkit can be understood as the latest iteration of prominent evidence repositories, which include the Campbell (Littell & White, 2018) and Cochrane (Levin, 2001) collaborations. At present, the Toolkit includes 23 strands that include a focus on tertiary prevention, including Focused Deterrence (Youth Endowment Fund, 2021), Restorative Justice (Skinner et al., 2025) and Pre-Court Diversion (Keenan et al., 2025).

Evidence repositories are necessary but not sufficient to ensure the use of evidence in policy and practice. Strategies such as seminars to present findings (Haynes et al., 2018), tailored briefings (Langer et al., 2016), and platforms for interactivity between researchers and policy makers (S. E. Brennan et al., 2016) all play an important role in increasing the use of evidence in policy and practice. The YEF has a series of Virtual Learning Cafes as part of the Evidence in Practice sessions. Typically, these are organised around a presentation about the Toolkit evidence on a particular topic, such as Functional Family Therapy (Youth Endowment Fund, 2026), alongside policy and practice experts who provide practical insights into the approach’s delivery. Evidence suggests that these types of meetings can be useful in generating demand for evidence and a useful way of bringing researchers and practitioners together, but are not sufficient to lead to behaviour change (Haynes et al., 2018; Langer et al., 2016).

YEF’s synthesis and dissemination functions are therefore complemented by a range of other initiatives to promote the use of evidence. This includes YEF’s practice guidance, which translates evaluation findings into actionable insights for frontline practitioners. For example, the Education, Children and Violence guidance (Youth Endowment Fund, 2024b) provides school, college and alternative provision (AP) leaders across England and Wales with five evidence-based recommendations to help prevent children’s involvement in violence, drawing on Toolkit evidence. YEF systems guidance supports commissioners and system leaders in making evidence-informed decisions about commissioning, scaling and integration across services. For example, the diversion systems guidance (Youth Endowment Fund, 2025a) makes several recommendations for improving support for children when they are arrested, focusing on the system for diverting children from formal criminal justice processes and outcomes. It includes a recommendation for improving access to therapy, given that large numbers of arrested children have unmet mental health needs (Samele et al., 2021). The recommendation is designed to redress the fact that very few receive any therapy, even though we know it is effective.

These guidance documents are supplemented with more intensive initiatives to support commissioners in becoming more evidence-informed. The Area Leaders Programme (Youth Endowment Fund, 2025c) is a programme for multi-agency violence reduction partnerships in local authority areas, that aims to equip area leaders with the knowledge, tools and partnerships needed to deliver effective, evidence-informed violence reduction. Each of the 10 sites completes a self-assessment framework to identify local strengths and areas for development. This forms the basis for 12 months of development, including engaging with a core set of modules for all sites, alongside tailored support to address site-specific priorities identified through the self-assessment.

Engagement with policymakers and commissioners is therefore not peripheral but integral to evaluation. Embedding dissemination, synthesis and implementation support alongside trial delivery increases the likelihood that evidence is not only generated, but used. Evidence from trials is actively disseminated through tailored briefings, regional learning events, partnerships with statutory agencies, and collaboration across the What Works network. By combining robust impact evidence with the implementation of learnings and practical guidance, YEF seeks to support more consistent, equitable and effective use of evidence within youth justice, policing, health and children’s services. In doing so, the Fund aims not only to identify what works to prevent youth violence but to increase the likelihood that high-quality evidence is used to improve policy and practice at scale.

In addition, a substantial and growing body of recent research and policy has explicitly cited and engaged with YEF’s research evidence syntheses and reports (Axford & Humayun, 2026; Bekaert et al., 2025; Bond-Taylor & Ellis, 2025; Denley et al., 2025; Farrington et al., 2022; Godwin et al., 2023; Hagen et al., 2025; Lewis et al., 2025; Marchment & Gill, 2025; Mercer et al., 2026; S. Moore et al., 2025; Skulberg et al., 2026; C. R. Smith et al., 2026; H. Smith et al., 2025; Stewart-Hall et al., 2023; Tura et al., 2025; UK Government, 2026; UK Home Office, 2026; Williams, 2025).^2^ This body of work demonstrates that YEF-commissioned evidence is being taken up beyond its original reporting context, informing wider evaluation practice, evidence synthesis and policy development.

In summary, YEF’s experience demonstrates that whilst evaluating tertiary prevention interventions presents substantial methodological and operational challenges, these challenges are surmountable. Through large-scale trials, methodological innovation and sustained investment in evidence mobilisation, YEF is contributing to a more robust and actionable evidence base to inform responses to serious youth violence in the UK.

## 7. Conclusions

This article has set out the practical realities of evaluating tertiary prevention interventions for children and young people involved in, or at high risk of, serious violence. It highlights both the importance of building a stronger evidence base in this area and the constraints that shape what is feasible in practice. Across the examples presented, a consistent message emerges: producing robust, decision-relevant evidence in tertiary contexts requires careful alignment between methodological ambition and the realities of systems and practice.

The core challenge is that tertiary evaluations take place in conditions where the need for rigour is highest, but the conditions for achieving it are the most challenging. Outcomes such as serious violence are relatively rare, even among high-risk groups, which creates substantial demands on sample size and follow-up data collection. At the same time, the populations targeted are often highly mobile, engaged with multiple services and experiencing instability, making recruitment, consent and retention more challenging. These issues are compounded by the need to work across complex, multi-agency systems where delivery and referral pathways are not always predictable or consistent. Taken together, these features mean that many common threats to evidence quality cannot be easily designed away, but must be anticipated and managed throughout the life of a study.

The experience discussed in this article suggests that maintaining rigour in this context depends as much on how evaluations are implemented as on how they are designed. Randomised controlled trials remain an important tool, but they need to be supported by pragmatic decisions about data sources, realistic assumptions about recruitment and attrition, and ongoing monitoring and adaptation during delivery. Approaches such as multi-site trials, the use of administrative data, and efforts to reduce participant burden reflect attempts to balance internal validity with feasibility in real-world settings.

More broadly, the article emphasises that evaluation in tertiary prevention is not a one-off technical exercise, but an ongoing process that requires coordination among funders, evaluators and delivery partners. It also highlights the importance of embedding considerations such as implementation fidelity, racial equity, and evidence use from the outset, rather than treating them as secondary concerns.

In summary, strengthening the evidence base for tertiary prevention will depend not only on testing more interventions, but on continuing to improve how evaluations are designed and delivered in challenging, real-world conditions.

## Figures and Tables

**Table 1:** Primary data and administrative data.

Data Type	Primary Data	Administrative Data
Examples	Standardised outcome measures administered to participants, e.g., Strengths and Difficulties Questionnaire	Hospital Episode Statistics collected by hospitals
	Qualitative interviews with participants	Crime recorded by police forces
Consent as an ethical basis for collecting and using personal data for the evaluation	Expected by ethics review boards as part of standard research practice	Not required
Consent as a legal basis for processing personal data for the evaluation	Not required	Not required
Other considerations and lessons in using the personal data for the evaluation	There needs to be security measures, control mechanisms and strong processes in place to minimise the risk of personal data breaches, disclosure and/or identifiability (UK Data Service, 2020).	
	Further mitigations to minimise these risks (and support ethics applications) include requiring minimal sensitive data or ‘special category data’ (Information Officer’s Office, 2025), minimising the amount and sensitivity of data and number of participants, and restricting access to personal data until after the delivery and follow-up period ends and analysis begins	Data sharing agreements need to be in place with relevant organisations

## Data Availability

The original contributions presented in this study are included in the article. Further inquiries can be directed to the corresponding author.

## Notes

1 We are grateful to an anonymous reviewer for the encouragement to reflect on this point.

2 These references have been established via forward citation of YEF reports.

## Abbreviations

The following abbreviations are used in this manuscript:

CYP	Children and Young People
RCT	Randomised Controlled Trial
YEF	Youth Endowment Fund

## Appendix A

The appendix summarises YEF’s evaluations of UK-developed tertiary prevention interventions.

**Project**	**Intervention Description**	**Cohort**	**Evaluation Design**	**Sample Size**
**Point of Arrest Diversion**				
REMEDI–Restorative Services	Restorative justice intervention delivered post-arrest by trained restorative mentors. Structured restorative conversations and mentoring over approximately 12 weeks.	10–17-year-olds arrested for violent or harmful behaviour. Tertiary prevention.	RCT (efficacy study)	348
Salford Foundation–STEER	Trauma-informed mentoring and wrap-around support delivered by trained youth workers. Weekly 1:1 mentoring plus family support over 6 months	10–17 identified by police/YOS as at risk of serious violence. Secondary prevention. Tertiary prevention	RCT (efficacy study)	654
REFRAME–We Are With You	Brief drug-focused diversion delivered in police custody. Two structured sessions delivered by specialist substance-misuse practitioners.	10–17 arrested for possession of controlled drugs. Secondary and tertiary prevention. Tertiary prevention	RCT (efficacy study)	388
**Hospital-based A&E navigators**				
Redthread Youth Violence Intervention	Youth workers embedded in A&E provide crisis support, brief intervention and referral following violence-related attendance.	11–25 presenting at A&E with violence-related injury. Secondary prevention.	QED (pilot/feasibility study)	1000
Multi-site Hospital Navigators Trial	Hospital-based navigators provide brief advice, emotional support and signposting across emergency departments.	Young people presenting with violence-related injury. Secondary prevention.	Feasibility study	N/A ^1^
PREVIP (South Wales)	Nurse-led and youth-worker-supported hospital intervention offering brief intervention and referral.	Emergency department patients with violence-related injuries, including young people. Secondary prevention.	Process/implementation evaluation	N/A ^2^
**Custody Based Diversion**				
Solution Focused Brief Therapy–Lancashire & South Cumbria NHS FT	Solution-Focused Brief Therapy delivered in police custody by trained clinicians. Up to four sessions.	10–17 in police custody. Tertiary prevention.	RCT (efficacy Study)	282
Divert Plus–Nottinghamshire VRU	Post-arrest diversion combining mentoring, speech and language therapy and personalised support.	10–17 arrested for violence-related offences. Tertiary prevention.	RCT (pilot study)	100
DIVERT Lambeth	Out-of-court disposal programme at point of arrest including mentoring and targeted support.	10–17 arrested in Lambeth. Secondary and tertiary prevention.	RCT (pilot study)	120
**Mentoring for at-risk and high-risk young people**				
United Borders	Music-based mentoring combining creative activity with structured mentoring over 12 months.	10–17 at risk of violence. Secondary prevention.	RCT (pilot study)	120
AudioActive (SHIFT)	Creative mentoring using music and arts alongside structured mentoring sessions over 12 months	Young people aged 10–17 at risk of violence. Secondary prevention. Tertiary Prevention	RCT (efficacy study, internal pilot)	586
SOS+ Programme	School-embedded mentoring delivered by mentors with lived experience over one academic year.	10–17 involved in criminal activity, violence or exploitation Tertiary Prevention	RCT (efficacy study)	960
Spark2Life–Meaningful Mentoring	Trauma-informed mentoring delivered weekly over 12 months.	11–18 involved in youth violence, gang activity, and/or crime as a perpetrator or victim Tertiary Prevention	RCT (efficacy study, internal pilot)	700
Media Academy Cymru (Cerridwen)	Media-based mentoring combining creative production with structured mentoring over 12 months.	Young people aged 10–17 at risk of violence. Secondary prevention.	RCT (efficacy study, internal pilot)	592
UpskillU–EXODUS mentoring programme	Mentoring and employability-focused support delivered over 12 months.	Young people aged 10–17 at risk of serious violence or have already offended. Secondary prevention. Tertiary prevention	RCT (efficacy study)	846
**Therapeutic projects**				
Your Choice	Cognitive-behavioural group intervention delivered in schools focusing on decision-making and peer influence.	11–18 assessed as medium or high risk of harm/vulnerability as a result of extrafamilial harm and has been considered by a multi-agency panel (typically MACE/Pre-MACE) Secondary prevention Tertiary prevention	Cluster RCT (efficacy study)	1658
Mentalization-Based Therapy for Conduct Disorder	Manualised mentalization-based therapy delivered by specialist clinicians in forensic CAMHS.	Children aged 8–16 with conduct disorder referred to FCAMHS. Tertiary prevention.	RCT (efficacy study)	632
^1^ The study did not proceed to the point where young people were recruited, so a sample size is not applicable. ^2^ The ethical and resource costs of working with such vulnerable patients in the NHS meant recruiting young patients this was not possible within the funded resource schedule.				

## Appendix B

The appendix summarises YEF’s evaluations of internationally developed interventions.

**Project**	**Intervention Description**	**Cohort**	**Evaluation Design**	**Sample Size**
Becoming a Man	Group-based CBT-informed programme delivered weekly by trained facilitators in schools over one academic year.	Boys aged 11–16 in high-risk schools. Secondary prevention. Tertiary prevention	Feasibility study	120
			Pilot Study	200
Functional Family Therapy–Extra Familial Harm	Systemic family therapy delivered by licenced FFT therapists over 3–5 months.	Young people aged 11–17 involved in serious violence or gang activity Tertiary prevention	Pilot Study	60
			Efficacy Study (RCT)	288
Focused Deterrence	Multi-agency deterrence model combining policing, community messaging and support services.	Individuals and groups at highest risk of serious violence Tertiary prevention	Efficacy Study (RCT)	2864
GenerationPMTO	Parenting programme delivered in group sessions by trained facilitators over ~14 weeks.	Parents of children aged 8–13 at risk of behavioural difficulties; Secondary prevention. Tertiary prevention	Feasibility study	80
			Pilot study	160
Multidimensional Family Therapy (MDFT)	Intensive manualised family therapy delivered by specialist clinicians over 4–6 months.	11–18 with substance misuse and behavioural problems; Tertiary prevention.	Efficacy Study (RCT)	400

## Appendix C

The appendix summarises YEF’s evaluations using the multi-site trial methodology.

**Project**	**Intervention Description**	**Cohort**	**Evaluation Design**	**Sample Size**
Mentoring multi-site trial	Mentoring for a minimum of 12 weeks duration. 12 sessions of at least 45 min over the course of 12 weeks. Adult paid mentors	Primarily targeting 10–14-year-olds (with some 15–17-year-olds) with risk factors related to youth violence. Secondary prevention.	Efficacy study	850
Towards Sport multi-site trial	24-week programme, with sports-based activities led by a paid coach	10–17-year-olds at a tertiary and secondary level of risk of offending	Efficacy Study (RCT)	3830
Moves Different Boxing multi-site trial	6-month group-based boxing programme delivered in local boxing clubs. Two one-hour sessions per week, delivered by trained coaches.	Young people in (or eligible to be in) academic years 9–13, who are at risk of involvement in youth violence and crime Secondary prevention Tertiary prevention	Efficacy Study (RCT)	3606

## References

[B1-behavsci-16-00626] Adler J. R., Edwards S., Scally M., Gill D., Puniskis M. J., Gekoski A., Horvath M. A. H. (2016). What works in managing young people who offend? A summary of the international evidence.

[B2-behavsci-16-00626] Andrews K., Parekh J., Peckoo S. (2019). A guide to incorporating a racial and ethnic equity perspective throughout the research process.

[B3-behavsci-16-00626] Anna Freud Centre (2022). Youth endowment fund outcomes framework.

[B4-behavsci-16-00626] Anna Freud Centre (2023). The youth endowment fund measures database for evaluations.

[B5-behavsci-16-00626] Asmussen K., Feinstein L., Martin J., Chowdry H. (2018). Foundations for life: What works to support parent-child interaction in the early years?.

[B6-behavsci-16-00626] Axford N., Berry V., Lloyd J., Hobbs T., Wyatt K. (2022a). Promoting learning from null or negative results in prevention science trials. Prevention Science.

[B7-behavsci-16-00626] Axford N., Berry V., Lloyd J., Wyatt K. (2022b). How can we optimise learning from trials in child and adolescent mental health?. Evidence Based Mental Health.

[B8-behavsci-16-00626] Axford N., Bywater T., Blower S., Berry V., Baker V., Morpeth L. (2017). Critical factors in the successful implementation of evidence-based parenting programmes. The wiley handbook of what works in child maltreatment.

[B9-behavsci-16-00626] Axford N., Humayun S. (2026). Preventing youth crime and violence: Intervention and evaluation issues. Behavioral Sciences.

[B10-behavsci-16-00626] Axford N., Tredinnick-Rowe J., Rybcyznska-Bunt S., Burns L., Green F., Thompson T. (2023). Engaging youth at risk of violence in services: Messages from research. Children and Youth Services Review.

[B11-behavsci-16-00626] Baidawi S., Ball R. (2023). Child protection and youth offending: Differences in youth criminal court-involved children by dual system involvement. Children and Youth Services Review.

[B12-behavsci-16-00626] Bandyopadhyay S. (2023). United Borders music mentoring programme; a pilot for a randomised controlled study.

[B13-behavsci-16-00626] Bandyopadhyay S., Evans E., Karavias Y., Menezes L. (2024). Remedi restorative mentors: A randomised controlled study.

[B14-behavsci-16-00626] Barnard M. (2024). Spark2Life’s meaningful mentoring programme to reduce the risk of violence and offending: A twoarmed randomised controlled trial with an internal pilot.

[B15-behavsci-16-00626] Beckman K. J., Jewett P. I., Gaҫad A., Borowsky I. W. (2024). Reducing re-arrest through community-led, police-initiated restorative justice diversion tailored for youth. Crime & Delinquency.

[B16-behavsci-16-00626] Bekaert S., Duman M., Cook G. (2025). The effectiveness of UK-based interventions to reduce school exclusion. A systematic review. Children and Youth Services Review.

[B17-behavsci-16-00626] Benson A. B., Pregler J. P., Bean J. A., Rademaker A. W., Eshler B., Anderson K. (1991). Oncologists’ reluctance to accrue patients onto clinical trials: An Illinois Cancer Center study. Journal of Clinical Oncology.

[B18-behavsci-16-00626] Bernal G., Adames C. (2017). Cultural adaptations: Conceptual, ethical, contextual, and methodological issues for working with ethnocultural and majority-world populations. Prevention Science.

[B19-behavsci-16-00626] Berry V., Melendez-Torres G. J., Axford N., Axberg U., de Castro B. O., Gardner F., Gaspar M. F., Handegård B. H., Hutchings J., Menting A., McGilloway S., Scott S., Leijten P. (2023). Does social and economic disadvantage predict lower engagement with parenting interventions? An integrative analysis using individual participant data. Prevention Science.

[B20-behavsci-16-00626] Bond-Taylor S., Ellis A. (2025). Rural marginality and violence affecting young people: Experiences from an English county. Journal of Youth Studies.

[B21-behavsci-16-00626] Boxford S., Jolliffe D., Clements S., Farrell J., Kaur K., Andersen E., Ramambason J., Morrison M., Butler S., de Souza C. (2026). Salford foundation’s Steer programme—A mentoring programme in Greater Manchester: Evaluation report.

[B22-behavsci-16-00626] Boxford S., Jolliffe D., Kaur K., Clements S., Morrison M. (2023). Randomised controlled trial of STEER.

[B23-behavsci-16-00626] Braga A. A., Weisburd D., Turchan B. (2019). Focused deterrence strategies effects on crime: A systematic review. Campbell Systematic Reviews.

[B24-behavsci-16-00626] Brennan I., McPherson T., Holmes E., Powrie M., Larigkou I., McFarlane P., Sutherland A., Graham W. (2026). Another chance fund focused deterrence programme: A multicentred randomised controlled trial.

[B25-behavsci-16-00626] Brennan I., Simanovic T., McFarlane P., Surtherland A., Graham W., Powrie M., Larigkou I., Holmes E. (2024). Focused deterrence randomised controlled trial: Early implementation report.

[B26-behavsci-16-00626] Brennan S. E., Cumpston M., Misso M. L., McDonald S., Murphy M. J., Green S. E. (2016). Design and formative evaluation of the policy liaison initiative: A long-term knowledge translation strategy to encourage and support the use of Cochrane systematic reviews for informing health policy. Evidence & Policy.

[B27-behavsci-16-00626] Brewster B., Robinson G., Silverman B. W., Walsh D. (2023). COVID-19 and child criminal exploitation in the UK: Implications of the pandemic for county lines. Trends in Organized Crime.

[B28-behavsci-16-00626] Bristow D., Carter L., Martin S. (2016). Using evidence to improve policy and practice: The UK what works centres. International and interdisciplinary insights into evidence and policy.

[B29-behavsci-16-00626] Brodie I., Day A. M., Kiff J., Osidipe T., Pitts J., Bateman T. (2025). Exploring racial disparity in diversion from the youth justice system in England and Wales.

[B30-behavsci-16-00626] Buckley P. R., Ebersole C. R., Steeger C. M., Michaelson L. E., Hill K. G., Gardner F. (2022). The role of clearinghouses in promoting transparent research: A methodological study of transparency practices for preventive interventions. Prevention Science.

[B31-behavsci-16-00626] Butt J. (2001). Partnership and power: The role of black and minority ethnic voluntary organisations in challenging racism. Partnership working.

[B32-behavsci-16-00626] Bywater T., Grindley N., Berry V., Blower S., Tobin K. (2019). The parent programme implementation checklist (ppic): The development and testing of an objective measure of skills and fidelity for the delivery of parent programmes. Child Care in Practice.

[B33-behavsci-16-00626] Cai Q., Chan A. C. Y., Lee S.-K., Marsalis S., Gewirtz A. H. (2022). Effectiveness of GenerationPMTO to promote parenting and child adjustment: A meta-analytic review. Clinical Child and Family Psychology Review.

[B34-behavsci-16-00626] Caridade S., Braga T. (2020). Youth Cyber Dating Abuse: A meta-analysis of risk and protective factors. Cyberpsychology: Journal of Psychosocial Research on Cyberspace.

[B35-behavsci-16-00626] Castro F. G., Barrera M., Martinez C. R. (2004). The cultural adaptation of prevention interventions: Resolving tensions between fidelity and fit. Prevention Science.

[B36-behavsci-16-00626] Cattan S., Bajpai P., Edbrooke-Childs J., Stapley E., Costa Dias M., Jacob J., Goodacre E., Labno A., Dhas V., Orchard E., Mckeaveney E., Khaling G., Kapoor A., New A., Rasul I. (2026). Your choice efficacy trial report.

[B37-behavsci-16-00626] Copeland L., Littlecott H., Couturiaux D., Hoddinott P., Segrott J., Murphy S., Moore G., Evans R. (2021). The what, why and when of adapting interventions for new contexts: A qualitative study of researchers, funders, journal editors and practitioners’ understandings. PLoS ONE.

[B38-behavsci-16-00626] Coulton S., Gannon T., Hendrie N. (2024a). Evaluation protocol: Re-frame: Randomised controlled efficacy trial of a diversion programme for adolescents in police custody who possess controlled drugs.

[B39-behavsci-16-00626] Coulton S., Hendrie N., Gannon T. (2026). Re-frame: Randomised controlled efficacy trial of a diversion programme for adolescents in police custody who possess illicit substances-trial report.

[B40-behavsci-16-00626] Coulton S., Hendrie N., Vass R., Gannon T., Wooton A., Rushworh-Claeys J., Sinetos J. (2024b). Randomized controlled internal pilot trial of a diversion programme for adolescents in police custody who possess illicit substances. Journal of Public Health.

[B41-behavsci-16-00626] Coulton S., Newbury-Birch D., Divers A., Hendrie N. (2025). Multidimensional family therapy to reduce alcohol and drug use in adolescents, an individually randomised controlled trial with embedded internal pilot—Evaluation protocol.

[B42-behavsci-16-00626] Damschroder L. J., Aron D. C., Keith R. E., Kirsh S. R., Alexander J. A., Lowery J. C. (2009). Fostering implementation of health services research findings into practice: A consolidated framework for advancing implementation science. Implementation Science.

[B43-behavsci-16-00626] Denley J., Ariel B., Assaraf N. (2025). Police-led interventions for deterring organized crime: A UK cluster randomized trial targeting criminal network recruiting and its vicarious effects on co-offenders. Justice Quarterly.

[B44-behavsci-16-00626] de Ribera O. S., Trajtenberg N., Shenderovich Y., Murray J. (2019). Correlates of youth violence in low- and middle-income countries: A meta-analysis. Aggression and Violent Behavior.

[B45-behavsci-16-00626] de Souza Leão L., Eyal G. (2019). The rise of randomized controlled trials (RCTs) in international development in historical perspective. Theory and Society.

[B46-behavsci-16-00626] DeWit D. J., DuBois D., Erdem G., Larose S., Lipman E. L. (2016). The role of program-supported mentoring relationships in promoting youth mental health, behavioral and developmental outcomes. Prevention Science.

[B47-behavsci-16-00626] Diderichsen F., Whitehead M., Dahlgren G. (2022). Planning for health equity in the crossfire between science and policy. Scandinavian Journal of Public Health.

[B48-behavsci-16-00626] Dixon J., Biehal N., Green J., Sinclair I., Kay C., Parry E. (2014). Trials and tribulations: Challenges and prospects for randomised controlled trials of social work with children. The British Journal of Social Work.

[B49-behavsci-16-00626] Dorling H., Cook A., Ollerhead L., Westmore M. (2015). The NIHR public health research programme: Responding to local authority research needs in the United Kingdom. Health Research Policy and Systems.

[B50-behavsci-16-00626] Durlak J. A. (2018). The fundamental importance of effective program implementation for successful character development. Journal of Character Education.

[B51-behavsci-16-00626] Durlak J. A., DuPre E. P. (2008). Implementation matters: A review of research on the influence of implementation on program outcomes and the factors affecting implementation. American Journal of Community Psychology.

[B52-behavsci-16-00626] Edbrooke-Childs J., Allcott-Watson H., Irvine K., Adler J., Stapley E., Busby A., Wellsted D., Stepanous J., Deighton J. (2025). A randomised controlled trial of mentalization-based therapy for conduct disorder for use with children referred to forensic child and adolescent mental health services.

[B53-behavsci-16-00626] Edovald T., Nevill C. (2021). Working out what works: The case of the education endowment foundation in England—Triin edovald, camilla nevill, 2021. ECNU Review of Education.

[B54-behavsci-16-00626] El-Enany N., Bruce-Jones E. (2015). Justice, resistance and solidarity: Race and policing in England and wales.

[B55-behavsci-16-00626] Ellison M., Cook W. (2025). Youth endowment fund: Administrative data guidance.

[B56-behavsci-16-00626] Ending Youth Violence Lab (n.d.). BIT.

[B57-behavsci-16-00626] Epstein D., Klerman J. A. (2012). When is a program ready for rigorous impact evaluation? The role of a falsifiable logic model. Evaluation Review.

[B58-behavsci-16-00626] Farrington D. P. (1996). Quantitative criminology in the United Kingdom in the 1990s: A brief overview. Journal of Quantitative Criminology.

[B59-behavsci-16-00626] Farrington D. P., Bergstrøm H., Jolliffe D. (2025). Childhood predictors of successful self-reported delinquents. Psychology, Crime & Law.

[B60-behavsci-16-00626] Farrington D. P., Gaffney H., Lösel F., Ttofi M. M. (2017). Systematic reviews of the effectiveness of developmental prevention programs in reducing delinquency, aggression, and bullying. Aggression and Violent Behavior, Systematic Reviews in Criminology.

[B61-behavsci-16-00626] Farrington D. P., Gaffney H., White H. (2022). Effectiveness of 12 types of interventions in reducing juvenile offending and antisocial behaviour. Canadian Journal of Criminology and Criminal Justice.

[B62-behavsci-16-00626] Farrington D. P., Jolliffe D., Coid J. (2021). Cohort profile: The Cambridge study in delinquent development (CSDD). Journal of Developmental and Life-Course Criminology.

[B63-behavsci-16-00626] Farrington D. P., Welsh B. C. (2003). Family-based prevention of offending: A meta-analysis. Australian & New Zealand Journal of Criminology.

[B64-behavsci-16-00626] Filges T., Rasmussen P. S., Andersen D., Jørgensen A.-M. K. (2015). Multidimensional family therapy (MDFT) for young people in treatment for non-opioid drug abuse: A systematic review. Campbell Systematic Reviews.

[B65-behavsci-16-00626] Fixsen D. L. (2005). Implementation research: A synthesis of the literature.

[B66-behavsci-16-00626] Fonagy P., Butler S., Cottrell D., Scott S., Pilling S., Eisler I., Fuggle P., Kraam A., Byford S., Wason J., Smith J. A., Anokhina A., Ellison R., Simes E., Ganguli P., Allison E., Goodyer I. M. (2020). Multisystemic therapy versus management as usual in the treatment of adolescent antisocial behaviour (START): 5-year follow-up of a pragmatic, randomised, controlled, superiority trial. The Lancet Psychiatry.

[B67-behavsci-16-00626] Forgatch M. S., Kjøbli J. (2016). Parent management training—Oregon model: Adapting intervention with rigorous research. Family Process.

[B68-behavsci-16-00626] Foundations (2025). Becoming a man.

[B69-behavsci-16-00626] Gaffney H., Farrington D. P., White H. (2021a). Boot camps toolkit technical report.

[B70-behavsci-16-00626] Gaffney H., Farrington D. P., White H. (2021b). Focussed deterrence toolkit technical report.

[B71-behavsci-16-00626] Gaffney H., Jolliffe D., White H. (2021c). Sports programmes Toolkit technical report.

[B72-behavsci-16-00626] Godwin J. V., Moore G., O’Reilly D., Hamilton M., Clift N., Moore S. C. (2023). Hospital-based violence prevention programmes in South Wales Emergency Departments: A process evaluation protocol. PLoS ONE.

[B73-behavsci-16-00626] Goodman R. (1997). The strengths and difficulties questionnaire: A research note—Goodman—1997. Journal of Child Psychology and Psychiatry.

[B74-behavsci-16-00626] Goodrum N. M., Cooper D. K., Edmunds S., Wippold G. M., Bradshaw J., Nguyen J. K., Milburn N., Are F. (2024). Achieving equity in child and adolescent mental health by addressing racism through prevention science. Adversity and Resilience Science.

[B75-behavsci-16-00626] Gottfredson D. C., Cook T. D., Gardner F. E. M., Gorman-Smith D., Howe G. W., Sandler I. N., Zafft K. M. (2015). Standards of evidence for efficacy, effectiveness, and scale-up research in prevention science: Next generation. Prevention Science: The Official Journal of the Society for Prevention Research.

[B76-behavsci-16-00626] Grant S., Wendt K. E., Leadbeater B. J., Supplee L. H., Mayo-Wilson E., Gardner F., Bradshaw C. P. (2022). Transparent, open, and reproducible prevention science. Prevention Science.

[B77-behavsci-16-00626] Green F., Axford N., Eastmond N., Berry V., Mannes J., Allen K., Callaghan L., Hobbs T. (2023a). Transporting an evidence-based youth development program to a new country: A narrative description and analysis of pre-implementation adaptation. Journal of Prevention.

[B78-behavsci-16-00626] Green F., Axford N., Harris J., Preece C., Mannes J., Callaghan L., Berry V., Santana de Lima E., Woodburn A. (2023b). Becoming a man (BAM): Feasibility study report.

[B79-behavsci-16-00626] Green F., Axford N., Preece C., Harris J., Allen K., Manzi S., Mannes J., Callaghan L., Berry V., Hobbs T., Santana de Lima E., Woodburn A. (2024). Becoming a man (BAM) pilot study report.

[B80-behavsci-16-00626] Gwata D., Ventriglio A., Hughes P., Deahl M. (2024). Youth violence and knife crime in ethnic minorities in the UK: A review of the literature. International Journal of Social Psychiatry.

[B81-behavsci-16-00626] Hagen T. L., Tan T. C. A., Thøgersen D. M., Bjørnebekk G. (2025). Functional family therapy across the COVID-19 pandemic. Frontiers in Psychology.

[B82-behavsci-16-00626] Hall A., Rowland J., Smith S., McNeil B., Purdon S., Bryson C., Peck S., Lewis J. (2023). Multi-site trial: Mentoring—Feasibility report.

[B83-behavsci-16-00626] Halpern D. (2015). What works: The rise of experimental government. Insisde the nudge unit.

[B84-behavsci-16-00626] Haylock S., Boshari T., Alexander E. C., Kumar A., Manikam L., Pinder R. (2020). Risk factors associated with knife-crime in United Kingdom among young people aged 10–24 years: A systematic review. BMC Public Health.

[B85-behavsci-16-00626] Haynes A., Rowbotham S. J., Redman S., Brennan S., Williamson A., Moore G. (2018). What can we learn from interventions that aim to increase policy-makers’ capacity to use research? A realist scoping review. Health Research Policy and Systems.

[B86-behavsci-16-00626] Heller S., Pollack H. A., Ander R., Ludwig J. (2013). Preventing youth violence and dropout: A randomized field experiment.

[B87-behavsci-16-00626] Heller S., Shah A. K., Guryan J., Ludwig J., Mullainathan S., Pollack H. A. (2017). Thinking, fast and slow? Some field experiments to reduce crime and dropout in Chicago. The Quarterly Journal of Economics.

[B88-behavsci-16-00626] Hobson J., Twyman-Ghoshal A., Banwell-Moore R., Ash D. P. (2022). Restorative justice, youth violence, and policing: A review of the evidence. Laws.

[B89-behavsci-16-00626] Humayun S., Herlitz L., Chesnokov M., Doolan M., Landau S., Scott S. (2017). Randomized controlled trial of functional family therapy for offending and antisocial behavior in UK youth. Journal of Child Psychology and Psychiatry.

[B90-behavsci-16-00626] Humayun S., Jolliffe D., Cleaver K. (2023a). Functional family therapy-gangs for young people at risk of child criminal exploitation and County Lines involvement.

[B91-behavsci-16-00626] Humayun S., Monks C., Jolliffe D. (2023b). Evaluation protocol: Efficacy randomised trial of functional family therapy—gangs.

[B92-behavsci-16-00626] Humphrey N., Hennessey A., Troncoso P., Panayiotou M., Black L., Petersen K., Wo L., Mason C., Ashworth E., Frearson K., Boehnke J. R., Pockett R., Lowin J., Foxcroft D., Wigelsworth M., Lendrum A. (2022). The good behaviour game intervention to improve behavioural and other outcomes for children aged 7–8 years: A cluster RCT. Public Health Research.

[B93-behavsci-16-00626] Hussain-Gambles M., Atkin K., Leese B. (2004). Why ethnic minority groups are under-represented in clinical trials: A review of the literature. Health and Social Care in the Community.

[B94-behavsci-16-00626] Hutchings J. M. (2012). Introducing, researching, and disseminating the incredible years programmes in Wales. International Journal of Conflict and Violence (IJCV).

[B95-behavsci-16-00626] Information Officer’s Office (2025). What is special category data?.

[B96-behavsci-16-00626] Irani M., Clements S., Boxford S., Jolliffe D., Kaur K., Morrison M. (2024). Media academy cymru’s (MAC) cerridwen project. A randomised controlled trial efficacy study with internal pilot.

[B97-behavsci-16-00626] Jones C. P. (2000). Levels of racism: A theoretic framework and a gardener’s tale. American Journal of Public Health.

[B98-behavsci-16-00626] Jull J., Whitehead M., Petticrew M., Kristjansson E., Gough D., Petkovic J., Volmink J., Weijer C., Taljaard M., Edwards S., Mbuagbaw L., Cookson R., McGowan J., Lyddiatt A., Boyer Y., Cuervo L. G., Armstrong R., White H., Yoganathan M., Welch V. (2017). When is a randomised controlled trial health equity relevant? Development and validation of a conceptual framework. BMJ Open.

[B99-behavsci-16-00626] Kane E., Cattell M. J., Durcan G., Parry M. J. (2025). Violence reduction, revisiting a public health approach. Public Health.

[B100-behavsci-16-00626] Keenan C., Hedges S., Mallion J. S., Allen K., Idres I., Murphy L., Hanratty J., Nugent R., Dunne C., Lyons F., Coady C., Towers J. (2025). Informal approaches to Pre-court diversion Toolkit technical report.

[B101-behavsci-16-00626] Kleeven A. T. H., Hilterman E. L. B., Mulder E. A., Popma A., de Vries Robbé M. (2025). Trajectories of justice involved youth: Changing risk and protective factors for violence. Youth Violence and Juvenile Justice.

[B102-behavsci-16-00626] Kovalenko A. G., Abraham C., Graham-Rowe E., Levine M., O’Dwyer S. (2022). What works in violence prevention among young people?: A systematic review of reviews. Trauma, Violence, & Abuse.

[B103-behavsci-16-00626] Kowalski M. A., Hamilton Z., Kigerl A., Baglivio M. T., Wolff K. T. (2023). Protecting against adversity: The role of positive childhood experiences in youth recidivism. Youth Violence and Juvenile Justice.

[B104-behavsci-16-00626] Krieger N. (2021). Structural racism, health inequities, and the two-edged sword of data: Structural problems require structural solutions. Frontiers in Public Health.

[B105-behavsci-16-00626] Krug E. G., Mercy J. A., Dahlberg L. L., Zwi A. B. (2002). The world report on violence and health. The Lancet.

[B106-behavsci-16-00626] Kumpfer K. L., Alvarado R., Smith P., Bellamy N. (2002). Cultural sensitivity and adaptation in family-based prevention interventions. Prevention Science.

[B107-behavsci-16-00626] Lakshminarayanan M., Skinner G., Li J., Tolan P., Du Bois D., White H. (2022). PROTOCOL: The effectiveness, implementation and cost effectiveness of mentoring programmes in reducing anti-social, violent and offending behaviour in children aged 17 years and below: A mixed method systematic review. Campbell Systematic Reviews.

[B108-behavsci-16-00626] Lalji R., Koh L., Francis A., Khalid R., Guha C., Johnson D. W., Wong G. (2024). Patient navigator programmes for children and adolescents with chronic diseases—Lalji, R—2024|Cochrane Library.

[B109-behavsci-16-00626] Lambie I., Allen R., Reil J., McLay J. (2026). Childhood offending, adverse experiences and systemic failure: Findings from a NZ national birth cohort 2000–2019. Children and Youth Services Review.

[B110-behavsci-16-00626] Lammy D. (2017). Lammy review: Final report.

[B111-behavsci-16-00626] Lange A. M. C., Humayun S., Jefford T. (2023). The feasibility of providing remote functional family therapy with adolescents during the COVID-19 pandemic: A mixed-method study. Child & Youth Care Forum.

[B112-behavsci-16-00626] Langer L., Tripney J. S., Gough D. (2016). The science of using science: Researching the use of research evidence in decision-making. (EPPI-Centre reports 3504). EPPI-centre, social science research unit, UCL institute of education: London, UK.

[B113-behavsci-16-00626] Lawson P. J., Flocke S. A. (2009). Teachable moments for health behavior change: A concept analysis. Patient Education and Counseling.

[B114-behavsci-16-00626] Lehmann P. S., Meldrum R. C. (2024). Disparities in youth arrest across racial and ethnic subgroups. Youth Violence and Juvenile Justice.

[B115-behavsci-16-00626] Levin A. (2001). The cochrane collaboration. Annals of Internal Medicine.

[B116-behavsci-16-00626] Leviton L. C., Trujillo M. D. (2017). Interaction of theory and practice to assess external validity. Evaluation Review.

[B117-behavsci-16-00626] Lewis J., Marsden S., Stefaniak A., Hewitt J. (2025). PROTOCOL: Non-criminal justice interventions for countering cognitive and behavioural radicalisation amongst children and adolescents: A systematic review of effectiveness and implementation. Campbell Systematic Reviews.

[B118-behavsci-16-00626] Lewis J., Smith S., McNeil B., McKaskill M., Purdon S., Bryson C. (2021). Multi-site trial: Feasibility study plan Mentoring.

[B119-behavsci-16-00626] Lipton B., Dickinson H., Bailie J., Hewitt B., Kavanagh A., Aitken Z., Shields M. (2025). Collaborating with young people: Identifying the barriers and facilitators in co-designed research. Health Expectations.

[B120-behavsci-16-00626] Littell J. H., White H. (2018). The campbell collaboration: Providing better evidence for a better world. Research on Social Work Practice.

[B121-behavsci-16-00626] Liu L., Zgoba K. M., Low S. (2024). Aggression and academic misconduct among justice-involved youth: The roles of facility environment, adverse childhood experiences and social competency. Youth Violence and Juvenile Justice.

[B122-behavsci-16-00626] Lord K., Hogan-Lloyd C., Boxford S. (2024). AudioActive’s Shift project. A randomised controlled trial efficacy study with internal pilot.

[B123-behavsci-16-00626] Macintyre S. (2011). Good intentions and received wisdom are not good enough: The need for controlled trials in public health. Journal of Epidemiology & Community Health.

[B124-behavsci-16-00626] Macintyre S., Petticrew M. (2000). Good intentions and received wisdom are not enough. Journal of Epidemiology & Community Health.

[B125-behavsci-16-00626] Magić J. (2023). Embedding mentors with lived experience in schools to reduce violent offending amongst children and young people: Randomised controlled efficacy trial of the SOS+ embedded mentoring programme.

[B126-behavsci-16-00626] Manful A., Willis R. (2024). Funding black-led micro-organisations in England. Voluntary Sector Review.

[B127-behavsci-16-00626] Marchment Z., Gill P. (2025). A comparison of project servator and routine stop and search outcomes in the city of London. Security Journal.

[B128-behavsci-16-00626] Martin J., Daly N., Wagstaff L., Forsyth E., McBride T. (2025). GenPMTO: Pilot study.

[B129-behavsci-16-00626] Matjasko J. L., Vivolo-Kantor A. M., Massetti G. M., Holland K. M., Holt M. K., Cruz J. D. (2012). A systematic meta-review of evaluations of youth violence prevention programs: Common and divergent findings from 25 years of meta-analyses and systematic reviews. Aggression and Violent Behavior.

[B130-behavsci-16-00626] Mbuagbaw L., Aves T., Shea B., Jull J., Welch V., Taljaard M., Yoganathan M., Greer-Smith R., Wells G., Tugwell P. (2017). Considerations and guidance in designing equity-relevant clinical trials. International Journal for Equity in Health.

[B131-behavsci-16-00626] McAra L., McVie S. (2007). Youth justice?: The impact of system contact on patterns of desistance from offending. European Journal of Criminology.

[B132-behavsci-16-00626] McBride T., Martin J., Wagstaff L., Abdi F., Smith S., Mills A. (2025). Moves different: Boxing to reduce youth offending. A two-armed pilot & efficacy randomised controlled trial.

[B133-behavsci-16-00626] Mercer G., Hunkin H., Figueroa F., Day A., Pilkington R. M., Malvaso C. G. (2026). Popular but poorly defined: A scoping review of youth diversion schemes. Australian Journal of Social Issues.

[B134-behavsci-16-00626] Ministry of Justice (2024). Statistics on ethnicity and the criminal justice system, 2024 (HTML).

[B135-behavsci-16-00626] Montgomery P., Chandan J. S. (2022). Redthread youth violence intervention programme evaluation.

[B136-behavsci-16-00626] Moore G., Campbell M., Copeland L., Craig P., Movsisyan A., Hoddinott P., Littlecott H., O’Cathain A., Pfadenhauer L., Rehfuess E., Segrott J., Hawe P., Kee F., Couturiaux D., Hallingberg B., Evans R. (2021). Adapting interventions to new contexts—The Adapt guidance. BMJ.

[B137-behavsci-16-00626] Moore S., Godwin J. V., Moore G., Hamilton M., O’Reilly D. (2025). Practitioner experiences of developing and implementing two UK ED-based hospital violence intervention programmes: A process evaluation. Emergency Medicine Journal.

[B138-behavsci-16-00626] Movsisyan A., Arnold L., Copeland L., Evans R., Littlecott H., Moore G., O’Cathain A., Pfadenhauer L., Segrott J., Rehfuess E. (2021). Adapting evidence-informed population health interventions for new contexts: A scoping review of current practice. Health Research Policy and Systems.

[B139-behavsci-16-00626] Munafò M., Neill J. (2016). Null is beautiful: On the importance of publishing null results. Journal of Psychopharmacology.

[B140-behavsci-16-00626] Murry V. M., Bradley C., Cruden G., Brown C. H., Howe G. W., Sepùlveda M.-J., Beardslee W., Hannah N., Warne D. (2024). Re-envisioning, retooling, and rebuilding prevention science methods to address structural and systemic racism and promote health equity. Prevention Science.

[B141-behavsci-16-00626] Naddaf M. (2025). AI is transforming peer review—And many scientists are worried. Nature.

[B142-behavsci-16-00626] Newman J., Cherney A., Head B. W. (2017). Policy capacity and evidence-based policy in the public service. Public Management Review.

[B143-behavsci-16-00626] NIHR (2025). The race equality framework: A practitioner’s guide for public involvement in research.

[B144-behavsci-16-00626] Nofi C. P., Roberts B. K., Cornell E., Tijerina M., Tussing O., Henry M. C., Sathya C. (2023). Hospital-based violence intervention programs to reduce firearm injuries in children: A scoping review. Journal of Pediatric Surgery.

[B145-behavsci-16-00626] Nutley S., Walter I., Davies H. T. O. (2009). Promoting evidence-based practice: Models and mechanisms from cross-sector review. Research on Social Work Practice.

[B146-behavsci-16-00626] Oakley A. (2006). Resistances to ‘new’ technologies of evaluation: Education research in the UK as a case study. Evidence & Policy.

[B147-behavsci-16-00626] OECD (2020). Building capacity for evidence-informed policy-making: Lessons from country experiences.

[B148-behavsci-16-00626] Oglesby-Neal A., Peterson B. (2021). Influence of race in the deep end of the juvenile justice system. Youth Violence and Juvenile Justice.

[B149-behavsci-16-00626] Oliver K., Cairney P. (2019). The dos and don’ts of influencing policy: A systematic review of advice to academics. Palgrave Communications.

[B150-behavsci-16-00626] Oliver K., Innvar S., Lorenc T., Woodman J., Thomas J. (2014). A systematic review of barriers to and facilitators of the use of evidence by policymakers. BMC Health Services Research.

[B151-behavsci-16-00626] Olver K., Cockbain E. (2021). Professionals’ views on responding to county lines-related criminal exploitation in the west midlands, UK. Child Abuse Review.

[B152-behavsci-16-00626] ONS (2025). Crime in England and wales—Office for national statistics.

[B153-behavsci-16-00626] Pardhan S., Sehmbi T., Wijewickrama R., Onumajuru H., Piyasena M. P. (2025). Barriers and facilitators for engaging underrepresented ethnic minority populations in healthcare research: An umbrella review. International Journal for Equity in Health.

[B154-behavsci-16-00626] Parmar A., Earle R., Phillips C. (2020). Race matters in criminology: Introduction to the Special Issue. Theoretical Criminology.

[B155-behavsci-16-00626] Peck A., Hutchinson M., Provost S. (2022). Evidencing predictors of adolescent to parent violence re-offending through linkage of police and health records. Youth Violence and Juvenile Justice.

[B156-behavsci-16-00626] Peguero A. A., Popp A. M. (2012). Youth violence at school and the intersection of gender, race, and ethnicity. Journal of Criminal Justice.

[B157-behavsci-16-00626] Petkovic J., Jull J., Yoganathan M., Dewidar O., Baird S., Grimshaw J. M., Johansson K. A., Kristjansson E., McGowan J., Moher D., Petticrew M., Robberstad B., Shea B., Tugwell P., Volmink J., Wells G. A., Whitehead M., Cuervo L. G., White H., Welch V. (2020). Reporting of health equity considerations in cluster and individually randomized trials. Trials.

[B158-behavsci-16-00626] Petrosino A., Turpin-Petrosino C., Hollis-Peel M. E., Lavenberg J. E. (2013). Scared straight and other juvenile awareness programs for preventing juvenile delinquency: A systematic review. Campbell Systematic Reviews.

[B159-behavsci-16-00626] Phillips C. (2011). Institutional racism and ethnic inequalities: An expanded multilevel framework. Journal of Social Policy.

[B160-behavsci-16-00626] Phillips C., Bowling B., Maguire M., Morgan R., Reiner R. (2017). Ethnicities, racism, crime and criminal justice. The Oxford handbook of criminology.

[B161-behavsci-16-00626] Piolanti A., Jouriles E. N., Foran H. M. (2022). Assessment of psychosocial programs to prevent sexual violence during adolescence: A systematic review and meta-analysis. JAMA Network Open.

[B162-behavsci-16-00626] Poorolajal J., Poorolajal J. (2025). Protocol deviation in randomized controlled trials. Illustrated epidemiology: A visual guide.

[B163-behavsci-16-00626] Prieto Curiel R., Collignon Delmar S., Bishop S. R. (2018). Measuring the distribution of crime and its concentration. Journal of Quantitative Criminology.

[B164-behavsci-16-00626] Rahal N., Wagstaff L., Daly N., Leith E., Wessel M., Martin J., McBride T. (2023). GenPMTO: Adaptation and feasibility study—Evaluation protocol.

[B165-behavsci-16-00626] Ramakrishnan R., Holland V., Agu N., Brady C., Marshall J. (2022). Characteristics associated with participant attrition and retention in a perinatal home visiting program. Prevention Science.

[B166-behavsci-16-00626] Raposa E. B., Rhodes J., Stams G. J. J. M., Card N., Burton S., Schwartz S., Sykes L. A. Y., Kanchewa S., Kupersmidt J., Hussain S. (2019). The effects of youth mentoring programs: A meta-analysis of outcome studies. Journal of Youth and Adolescence.

[B167-behavsci-16-00626] Rasul I., Cattan S., Costa Dias M., Edbrooke-Childs J. (2021). The london young people study: Evaluation of the your choice programme using a cluster randomised controlled trial.

[B168-behavsci-16-00626] Redwood S., Gill P. S. (2013). Under-representation of minority ethnic groups in research—Call for action. British Journal of General Practice.

[B169-behavsci-16-00626] Roberts L., Tamene M., Orta O. R. (2018). The intersectionality of racial and gender discrimination among teens exposed to dating violence. Ethnicity & Disease.

[B170-behavsci-16-00626] Roberts S. O., Bareket-Shavit C., Dollins F. A., Goldie P. D., Mortenson E. (2020). Racial inequality in psychological research: Trends of the past and recommendations for the future. Perspectives on Psychological Science.

[B171-behavsci-16-00626] Robling M., Bekkers M.-J., Bell K., Butler C. C., Cannings-John R., Channon S., Martin B. C., Gregory J. W., Hood K., Kemp A., Kenkre J., Montgomery A. A., Moody G., Owen-Jones E., Pickett K., Richardson G., Roberts Z. E. S., Ronaldson S., Sanders J., Torgerson D. (2016). Effectiveness of a nurse-led intensive home-visitation programme for first-time teenage mothers (Building Blocks): A pragmatic randomised controlled trial. The Lancet.

[B172-behavsci-16-00626] Rooney R. M., Hopkins A., Peckover J., Coleman K., Sampson R., Alati R., Hassan S., Pollard C. M., Dantas J. A. R., Lobo R., Jeemi Z., Burns S., Cunningham R., Monterosso S., Millar L., Dovchin S., Oliver R., Bhoyroo R., Ayano G. (2024). Protective factors, risk factors, and intervention strategies in the prevention and reduction of crime among adolescents and young adults aged 12–24 years: A scoping review protocol. PLoS ONE.

[B173-behavsci-16-00626] Rowland J., Quail H., Fischer J., Hall A., Purdon S., Bryson C., Mahmood Z., McNeil B., Verdugo P., Baan A.-M., Lewis J. (2024). Multi-site trial: Short-term mentoring—Efficacy trial report.

[B174-behavsci-16-00626] Samele C., McKinnon I., Brown P., Srivastava S., Arnold A., Hallett N., Forrester A. (2021). The prevalence of mental illness and unmet needs of police custody detainees. Criminal Behaviour and Mental Health.

[B175-behavsci-16-00626] Sampson R. J., Neil R. (2024). The social foundations of racial inequalities in arrest over the life course and in changing times. Criminology: An Interdisciplinary Journal.

[B176-behavsci-16-00626] Shamrova D., Tolchinsky J., Boppre B., Alberton A., Williams J. (2025). The perspectives of Black youth on risk and protective factors for peer involvement in the juvenile legal system. Child and Adolescent Social Work Journal.

[B177-behavsci-16-00626] Sherman L. W., Strang H., Mayo-Wilson E., Woods D. J., Ariel B. (2015). Are restorative justice conferences effective in reducing repeat offending? Findings from a Campbell systematic review. Journal of Quantitative Criminology.

[B178-behavsci-16-00626] Simanovic T., McFarlane P., Brennan I., Graham W., Sutherland A. (2024). Focused deterrence: A protocol for a realist multisite randomised controlled trial for evaluating a violence prevention intervention in the UK. PLoS ONE.

[B179-behavsci-16-00626] Skinner G., Jolliffe D., Gaffney H., Ferreira J., Eggins E., Higginson A. (2025). The effect of restorative justice interventions for children and young people on offending and reoffending: A mixed-methods systematic review.

[B180-behavsci-16-00626] Skivington K., Matthews L., Simpson S. A., Craig P., Baird J., Blazeby J. M., Boyd K. A., Craig N., French D. P., McIntosh E., Petticrew M., Rycroft-Malone J., White M., Moore L. (2021). A new framework for developing and evaluating complex interventions: Update of Medical Research Council guidance. BMJ.

[B181-behavsci-16-00626] Skulberg K., Tan T. C. A., Thøgersen D. M., Taraldsen K., Bjørnebekk G. (2026). Multisystemic therapy for adolescents through the COVID-19 pandemic in Norway. Journal of Child and Family Studies.

[B182-behavsci-16-00626] Smith C. R., Harris J., Quigg Z. A. (2026). Evaluation of the merseyside navigator programme: A hospital-based violence intervention programme for young people affected by or at risk of violence. Children and Youth Services Review.

[B183-behavsci-16-00626] Smith D. J., McVie S. (2003). Theory and method in the edinburgh study of youth transitions and crime. The British Journal of Criminology.

[B184-behavsci-16-00626] Smith H., Deakin J., Lennox C., Ruding S. (2025). The effects of arts and culture programmes on youth crime: A rapid review.

[B185-behavsci-16-00626] Spyropoulas N., Somani R., Mousteri V., McKnight L. (2025). Toward sport—A randomised multisite trial to evaluate a sports-based intervention aiming to enhance postive outcomes for children and young people in the context of youth offending.

[B186-behavsci-16-00626] Stanford M., Gilbert L., Blackshaw E., Johnson M., Bhalla N., Lawrence H. (2024). Efficacy evaluation of the EXODUS mentoring programme.

[B187-behavsci-16-00626] Stewart-Hall C., Langham L., Miller P. (2023). Preventing school exclusions of black children in England—A critical review of prevention strategies and interventions. Equity in Education & Society.

[B188-behavsci-16-00626] Strang H., Sherman L. W., Mayo-Wilson E., Woods D., Ariel B. (2013). Restorative justice conferencing (RJC) using face-to-face meetings of offenders and victims: Effects on offender recidivism and victim satisfaction. A systematic review. Campbell Systematic Reviews.

[B189-behavsci-16-00626] Sutherland A., Makinson L., Bisserbe C., Farrington J. (2023). Hospital navigators: Multi-site evaluation of practices.

[B190-behavsci-16-00626] Šimkovic M., Träuble B. (2019). Robustness of statistical methods when measure is affected by ceiling and/or floor effect. PLoS ONE.

[B191-behavsci-16-00626] Themelidis L., Lorenzo-Dus N. (2025). Child-to-child harmful online sexual exchanges: A systematic review of the literature. Safer Communities.

[B192-behavsci-16-00626] The Young Foundation (2021). Far to go: Diversity and inclusion in UK social research.

[B193-behavsci-16-00626] Thierry K. L., Vincent R. L., Norris K. (2022). Teacher-level predictors of the fidelity of implementation of a social-emotional learning curriculum. Early Education and Development.

[B194-behavsci-16-00626] Thompson P., Playle R. (2025). Solution Focused brief therapy (SFBT) in 10–17-year-olds presenting at police custody: Statistical Analysis Plan.

[B195-behavsci-16-00626] Tilki M., Thompson R., Robinson L., Bruce J., Chan E., Lewis O., Chinegwundoh F., Nelson H. (2015). The BME third sector: Marginalised and exploited. Voluntary Sector Review.

[B196-behavsci-16-00626] Tolan P., Henry D., Schoeny M., Bass A., Lovegrove P., Nichols E. (2013). Mentoring interventions to affect juvenile delinquency and associated problems: A systematic review. Campbell Systematic Reviews.

[B197-behavsci-16-00626] Tura F., Weir R., Blom N., Adeniyi O. (2025). Increases in disadvantage and instability are associated with rising violence. The British Journal of Criminology.

[B198-behavsci-16-00626] UK Data Service (2020). What is the Five Safes framework?.

[B199-behavsci-16-00626] UK Government (2026). Protecting lives, building hope: A plan to halve knife crime.

[B200-behavsci-16-00626] UK Home Office (2026). Freedom from violence and abuse: A cross-government strategy.

[B201-behavsci-16-00626] Ullman R., Lereya S. T., Glendinnin F., Deighton J., Labno A., Liverpool S., Edbrooke-Childs J. (2024). Constructs associated with youth crime and violence amongst 6–18 year olds: A systematic review of systematic reviews. Aggression and Violent Behavior.

[B202-behavsci-16-00626] Uwadiae F., Rutherford D., Medupin T., Woods A., Lewis-Wilson S. (2026). From surviving to thriving: Strengthening black-led initiatives. Wellcome Open Res.

[B203-behavsci-16-00626] Vallance P., MacKillop E., Downe J., Notman G. (2025). Understanding the impact of knowledge brokering organizations: The case of the UK What Works Centres. Research Evaluation.

[B204-behavsci-16-00626] Van Aert R. C., Wicherts J. M., Van Assen M. A. (2019). Publication bias examined in meta-analyses from psychology and medicine: A meta-meta-analysis. PLoS ONE.

[B205-behavsci-16-00626] Walcott S., Nightingale G. (2025). How racism affects health.

[B206-behavsci-16-00626] Walker D., Infante A. A., Knight D. (2022). Examining the impact of mental health, substance use, and co-occurring disorders on juvenile court outcomes. Journal of Research in Crime and Delinquency.

[B207-behavsci-16-00626] Walshe C., Algorta G. P., Dodd S., Hill M., Ockenden N., Payne S., Preston N. (2016). Protocol for the end-of-life social action study (ELSA): A randomised wait-list controlled trial and embedded qualitative case study evaluation assessing the causal impact of social action befriending services on end of life experience. BMC Palliative Care.

[B208-behavsci-16-00626] Wandersman A., Imm P., Chinman M., Kaftarian S. (2000). Getting to outcomes: A results-based approach to accountability. Evaluation and Program Planning.

[B209-behavsci-16-00626] Ward V., House A., Hamer S. (2009). Knowledge brokering: The missing link in the evidence to action chain?. Evidence & Policy.

[B210-behavsci-16-00626] Welsh B. C., Farrington D. P., Welsh B. C., Farrington D. P. (2007). Evidence-based crime prevention. Preventing crime: What works for children, offenders, victims and places.

[B211-behavsci-16-00626] White H. (2019). The twenty-first century experimenting society: The four waves of the evidence revolution. Palgrave Communications.

[B212-behavsci-16-00626] Wilkinson D., Chopra I., Badger S. (2024). A systematic review of evidence capturing efficacy of community and school-based approaches to knife crime intervention and prevention programs. Journal of Criminal Psychology.

[B213-behavsci-16-00626] Williams S. C. (2025). What are the experiences of those engaged in professional youth work in a formal education college in the UK?. Youth.

[B214-behavsci-16-00626] Wilson D. B., Lipsey M. W. (2024). Scaling up effective juvenile delinquency programs by focusing on change levers: Evidence from a large meta-analysis. Criminology & Public Policy.

[B215-behavsci-16-00626] Wiltsey Stirman S., Baumann A. A., Miller C. J. (2019). The FRAME: An expanded framework for reporting adaptations and modifications to evidence-based interventions. Implementation Science.

[B216-behavsci-16-00626] Wolff N. (2000). Using randomized controlled trials to evaluate socially complex services: Problems, challenges and recommendations. The Journal of Mental Health Policy and Economics.

[B217-behavsci-16-00626] Wong J. S., Lee C., Beck N. (2024). The effects of aftercare/resettlement services on crime and violence in children and youth: A systematic review. Campbell Systematic Reviews.

[B218-behavsci-16-00626] Wortley E., Hagell A. (2021). Young victims of youth violence: Using youth workers in the emergency department to facilitate ‘teachable moments’ and to improve access to services. Archives of Disease in Childhood-Education and Practice.

[B219-behavsci-16-00626] Wyatt K. A., Bell J., Cooper J., Constable L., Siero W., Pozo Jeria C., Darling S., Smith R., Hughes E. K. (2024). Involvement of children and young people in the conduct of health research: A rapid umbrella review. Health Expectations.

[B220-behavsci-16-00626] YEF (2022). The home office and youth endowment fund invest £7 million in focused deterrence.

[B221-behavsci-16-00626] Youth Endowment Fund (n.d.). Evaluator panel.

[B222-behavsci-16-00626] Youth Endowment Fund (2021). Focused deterrence|YEF toolkit.

[B223-behavsci-16-00626] Youth Endowment Fund (2022). Youth mentoring programmes|YEF toolkit.

[B224-behavsci-16-00626] Youth Endowment Fund (2023). Data protection information for YEF evaluations: Guidance for projects and evaluators.

[B225-behavsci-16-00626] Youth Endowment Fund (2024a). Children, violence and vulnerability 2024.

[B226-behavsci-16-00626] Youth Endowment Fund (2024b). Education, Children and Violence: Guidance for school, college and alternative provision leaders to help prevent children’s involvement in violence.

[B227-behavsci-16-00626] Youth Endowment Fund (2025a). Diversion practice guidance: Guidance on how to deliver diversion effectively for children and young people.

[B228-behavsci-16-00626] Youth Endowment Fund (2025b). Focused deterrence delivery guidance.

[B229-behavsci-16-00626] Youth Endowment Fund (2025c). Nine local authorities join new programme to strengthen multi-agency violence prevention.

[B230-behavsci-16-00626] Youth Endowment Fund (2025d). Race Equity in YEF’s research and evaluation.

[B231-behavsci-16-00626] Youth Endowment Fund (2025e). Racial disproportionality in violence affecting children and young people.

[B232-behavsci-16-00626] Youth Endowment Fund (2025f). The Youth Endowment Fund’s approach to race equity.

[B233-behavsci-16-00626] Youth Endowment Fund (2025g). Youth endowment fund: Race equity progress report 2025.

[B234-behavsci-16-00626] Youth Endowment Fund (2026). Functional family therapy: Evidence and practice.

